# Synthetic Heparan Sulfate Oligosaccharides Inhibit Endothelial Cell Functions Essential for Angiogenesis

**DOI:** 10.1371/journal.pone.0011644

**Published:** 2010-07-21

**Authors:** Claire L. Cole, Steen U. Hansen, Marek Baráth, Graham Rushton, John M. Gardiner, Egle Avizienyte, Gordon C. Jayson

**Affiliations:** 1 School of Cancer and Enabling Sciences, The University of Manchester, Manchester, United Kingdom; 2 School of Chemistry and Manchester Interdisciplinary Biocentre, The University of Manchester, Manchester, United Kingdom; 3 Institute of Chemistry, Centre of Glycomics, Slovak Academy of Sciences, Bratislava, Slovakia; National Cancer Institute, United States of America

## Abstract

**Background:**

Heparan sulfate (HS) is an important regulator of the assembly and activity of various angiogenic signalling complexes. However, the significance of precisely defined HS structures in regulating cytokine-dependent angiogenic cellular functions and signalling through receptors regulating angiogenic responses remains unclear. Understanding such structure-activity relationships is important for the rational design of HS fragments that inhibit HS-dependent angiogenic signalling complexes.

**Methodology/Principal Findings:**

We synthesized a series of HS oligosaccharides ranging from 7 to 12 saccharide residues that contained a repeating disaccharide unit consisting of iduronate 2-*O*-sulfate linked to glucosamine with or without *N*-sulfate. The ability of oligosaccharides to compete with HS for FGF2 and VEGF_165_ binding significantly increased with oligosaccharide length and sulfation. Correspondingly, the inhibitory potential of oligosaccharides against FGF2- and VEGF_165_-induced endothelial cell responses was greater in longer oligosaccharide species that were comprised of disaccharides bearing both 2-*O*- and *N*-sulfation (2SNS). FGF2- and VEGF_165_-induced endothelial cell migration were inhibited by longer 2SNS oligosaccharide species with 2SNS dodecasaccharide activity being comparable to that of receptor tyrosine kinase inhibitors targeting FGFR or VEGFR-2. Moreover, the 2SNS dodecasaccharide ablated FGF2- or VEGF_165_-induced phosphorylation of FAK and assembly of F-actin in peripheral lamellipodia-like structures. In contrast, FGF2-induced endothelial cell proliferation was only moderately inhibited by longer 2SNS oligosaccharides. Inhibition of FGF2- and VEGF_165_-dependent endothelial tube formation strongly correlated with oligosaccharide length and sulfation with 10-mer and 12-mer 2SNS oligosaccharides being the most potent species. FGF2- and VEGF_165_-induced activation of MAPK pathway was inhibited by biologically active oligosaccharides correlating with the specific phosphorylation events in FRS2 and VEGFR-2, respectively.

**Conclusion/Significance:**

These results demonstrate structure-function relationships for synthetic HS saccharides that suppress endothelial cell migration, tube formation and signalling induced by key angiogenic cytokines.

## Introduction

Heparan sulfate (HS), a member of glycosaminoglycan (GAG) family, exists as a covalently linked component of cell surface or extracellular matrix core proteins forming proteoglycans, integral components of several cell surface receptor signalling complexes [1]–[3]. HS is composed of alternate hexuronic acid and N-substituted glucosamine residues. Following polymerisation modifications include C5 epimerisation of glucuronic acid to form iduronate and variable sulfation at the 2-*O* position of iduronic acid and 6-*O*-, 3-*O*- and *N*-positions of glucosamine creating highly sulfated HS domains separated by poorly sulfated, glucuronate-containing regions [1]–[2].

The highly sulfated HS regions (S-domains) facilitate numerous signalling events by interacting with a number of growth factors, chemokines and their receptors [1]–[3]. Several studies suggest that some growth factors interact with specific sequences within HS S-domains that are required for both binding and biological activity [4]–[8]. For example, 2-*O*-sulfated iduronate and N-sulfated glucosamine are essential for the interaction between FGF2 and HS, whereas 6-*O*-sulfate groups are required for mitogenic activity of the complex [4]–[6]. FGF1 requires 2-*O*-, *N*- and 6-*O*-sulfate groups for optimal binding to HS and mitogenesis [7]. On the other hand, binding of a VEGF_165_ dimer to HS requires two highly sulfated HS domains linked through a partially sulfated transitional sequence [8]. Sulfates at 2-*O*-, 6-*O*- and *N*-positions significantly contribute to the binding [8]. Some growth factors require organized HS domains rather than defined sulfation patterns [9]. For example, platelet-derived growth factor B (PDGF-B), which guides pericyte recruitment during vascular development, binds HS with an affinity that depends on the spacing of *N*-sulfated domains in HS chains [9].

Since such diverse, although specific, interactions between HS and angiogenic cytokines are critical in modulating signalling through respective receptors, angiogenic cytokine-receptor-HS complexes represent a putative target for novel inhibitors of pathophysiological angiogenesis. One approach could be to develop HS oligosaccharides that competitively inhibit the biological activity of membranous HS proteoglycans preventing signalling through the cognate receptor. Indeed the potential therapeutic benefit of low molecular weight heparin (LMWH), a highly sulfated HS, was observed in pre-clinical models where it acted as an antiangiogenic, antimetastatic, and anti-inflammatory agent [10]–[12]. Moreover, in an *in vivo* angiogenesis assays LMWH-derived octa- and deca-saccharides significantly reduced microvessel density in response to FGF2 [13].

Despite attempts to generate pure length-defined heparin oligosaccharides, chromatographic preparations represent a heterogeneously sulfated population of oligosaccharides, thus obscuring the critical structural features of HS/heparin required to inhibit different angiogenic cytokines. Using a chemical synthesis strategy we have generated a series of HS oligosaccharides with a defined number of saccharide residues, where the component disaccharides contained iduronate 2-*O*-sulfate alone or the same moiety with glucosamine *N*-sulfate. A strong correlation was observed between the structure of oligosaccharides, their affinity to key angiogenic cytokines and biological activity in targeting specific cytokine-dependent endothelial cell functions.

## Materials and Methods

### Chemical synthesis

The synthesis was performed using traditional solution phase chemistry and after each step the products were purified by either crystallization or flash column chromatography using Silicagel 60 (Fluka, Gillingham, UK). The final products were purified by size exclusion chromatography using Sephadex G-25 (Sigma-Aldrich, Gillingham, UK) and lyophilised. For each step the product structure and purity was confirmed by NMR spectroscopy and mass spectrometry. Reactants, starting materials and solvents were purchased from Sigma-Aldrich, Fluka and Alfa-Aesar (Heysham, UK) and used without further purification.

### Cell culture

Primary human umbilical vein endothelial cells (HUVECs) were purchased from Lonza (Slough, UK) and routinely cultured in EBM-2 medium supplemented with SingleQuots growth supplements (Lonza) up to passage 7. SV40 immortalized HUVEC cell line (EVLC2) was a gift from Prof. C. Dive (Paterson Institute for Cancer Research, Manchester, UK). EVLC2 cells were maintained in Dulbecco's Modified Eagle Medium containing F12 nutrient mixture, L-glutamine and pyruvate (DMEM; Invitrogen, Paisley, UK) and supplemented 10% foetal bovine serum (FBS; Promega, Southampton, UK). Normal human dermal fibroblasts (NHDFs) were purchased from Lonza and cultured in DMEM/F12 medium containing L-glutamine and pyruvate (Invitrogen) and supplemented with 10% FBS (Promega).

### HS competition assays

Ninety six well heparin-binding plates (Iduron, Manchester, UK) were coated with heparin sulfate (HS; Iduron) at 25 µg/ml in standard assay buffer (SAB; 50 mM sodium acetate pH 7.3, 150 mM sodium chloride and 0.2% Tween20) for 24 hours, washed 3 times with SAB and incubated with 20 mg/ml BSA diluted in SAB for 5 hours. After 3 washes with SAB, 10 ng of each cytokine was diluted in 100 µl SAB plus 10 mg/ml BSA. Oligosaccharides were mixed at 0.01–10 µg/ml concentration range for FGF2 or 0.1–100 µg/ml range for VEGF_165_ (all from R&D Systems, Abingdon, UK). Cytokine and oligosaccharide mixtures were added to immobilized HS for 2 hours. Wells were washed 3 times with SAB and biotin-tagged primary antibodies against FGF2 (R&D Systems, DuoSet ELISA Development kit, #DY233) or VEGF_165_ (R&D Systems, #BAF 293) diluted 1∶200 in SAB plus BSA were added to the wells. After 2 hour incubation wells were washed 3 times with SAB and streptavidin-HRP (R&D Systems, #890803) diluted 1∶200 in SAB plus BSA was added for 30 min at room temperature. Wells were washed 3 times with SAB and then incubated for 20 min in TMB (Sigma-Aldrich). The reaction was stopped with 2 M sulphuric acid and optical density measured at 450 nm.

### Sulforhodamine B (SRB) proliferation assay

HUVECs were plated at 1250 cells/well in 24 well plates in 250 µl of EBM-2 medium without SingleQuots growth supplements plus 1% FBS and placed in the cell culture incubator for 6 hours before dosing. One hour before dosing oligosaccharides were mixed with FGF2 (R&D Systems, 20 ng/ml) or VEGF_165_ (R&D Systems, 20 ng/ml) at 50 µg/ml (corresponding molar concentration for each oligosaccharide is shown in Table 1) and incubated at 37°C and then added to the cells for 96 hours. Cells were fixed with 5% trichloroacetic acid (TCA) for 1 hour at +4°C, washed with water and allowed to dry. Cells were stained with 4% sulforhodamine B in 1% acetic acid for 30 min, excess stain was removed by washing in 1% acetic acid and wells were allowed to dry. Stain was solubilised in 200 µl 10 mM Tris pH 8.8 and transferred to 96-well plate for reading on a microplate reader at 540 nm.

**Table 1:** Synthetic oligosaccharide series.

Oligosaccharide ength	Sulfation*	Molecular weight (g/mol)	Molar dose (µM) for 10 µg/ml	Molar dose (µM) for 50 µg/ml
7-mer	2S	1577	6.3	31.5
7-mer	2SNS	1985	5.0	25.0
8-mer	2S	1979	5.1	25.5
8-mer	2SNS	2387	4.2	21.0
9-mer	2S	2038	4.9	24.5
9-mer	2SNS	2548	3.9	19.6
10-mer	2S	2441	4.1	20.5
10-mer	2SNS	2951	3.4	16.9
12-mer	2S	2800	3.6	18.0
12-mer	2SNS	3412	2.9	14.7

*2S - iduronic acid 2-*O*-sulfate; 2SNS - iduronic acid 2-*O*-sulfate (2S) and glucosamine *N*-sulfate (NS).

### Wound-healing assay

EVLC2 cells were seeded in 96-well plate at 2×10^4^ cells per well and incubated for 16 hours at +37°C in a cell culture incubator. Confluent monolayers were serum-starved in EBM-2 medium lacking SingleQuots growth supplements and containing 0.1% FBS for 24 hours and the wound was created by manually scraping cell monolayer with a pipette tip. Cells were washed and incubated with either EBM-2 medium without SingleQuots growth supplements plus 0.1% FBS or medium supplemented with FGF2 (20 ng/ml), VEGF_165_ (20 ng/ml), EGF (20 ng/ml) or VEGF_121_ (20 ng/ml) (all from R&D Systems). Oligosaccharides were pre-incubated with the cytokines before adding to cells for 30 min at room temperature. Maximal concentration of synthetic oligosaccharides in would-healing assay was 50 µg/ml (equivalent molar concentration for each oligosaccharide is shown in Table 1). Heparin, dermatan sulfate 4-*O*-sulfated (DS 4S) 12-mer and a mixture of chondroitin sulfate 4-*O*- or 6-*O*-sulfated (CS 4S/6S) 12-mers were dosed at 50 µg/ml concentration. FGFR inhibitor PD173074 (Sigma, # P2499) and VEGFR-2 inhibitor SU4312 (Tocris Bioscience, Bristol, UK, # 1459) were used at 0.2 µM and 0.4 µM concentration, respectively. To determine IC_50_ values oligosaccharides were used at 0.04, 0.2, 1, 5, 25 and 50 µg/ml concentrations (molar concentrations are shown in Table 1). Each treatment was performed in triplicate. Phase contrast images were taken immediately after adding cytokines with or without oligosaccharides and after 24 hours of incubation using Zeiss Axiovert 200 M microscope (Zeiss, Hertfordshire, UK) enclosed in a full environmental chamber. The unpopulated areas were analysed using MetaMorph image analysis software (Molecular Devices, Uckfield, UK) by measuring unpopulated area at 0 and 24 hours and cell advancement area was derived for each treatment. IC_50_ values were produced using GraphPad Prism software (GraphPad Software, La Jolla, CA, USA).

### Immunofluorescence microscopy

Cells were fixed with 4% paraformaldehyde in Tris-buffered saline (TBS) for 10 min, permeabilized with TBS containing 0.5% Triton X-100 for 15 min, and blocked with 5% FBS in TBS for an hour. Cells were incubated with polyclonal rabbit anti-phospho-FAK (pTyr397) (Invitrogen, #44–625G) antibody diluted 1∶100 overnight at +4°C. Cells were washed and incubated with AlexaFluor488–conjugated donkey anti-rabbit IgG antibody (1∶1000; Invitrogen, #A11034) and AlexaFluor568 phalloidin (1∶40; Invitrogen, #A12380) to visualize F-actin for 1 hour at room temperature. After washing samples were analysed by fluorescence microscopy at ×20 magnification (Solent Scientific, Segensworth, UK).

### Tube formation assay

HUVECs (500 cells/bead) were mixed with Cytodex 3 microcarriers (Amersham Pharmacia Biotech, Bucks, UK) in 1 ml EBM-2 medium supplemented with SingleQuots growth supplements. The bead-cell mixture was placed at 37°C in cell culture incubator and shaken gently every 20 min for 4 hours. The mixture was then placed in a 10 mm tissue culture dish in 4 ml EBM-2 medium and incubated overnight. The following morning the beads were harvested, washed 3 times with 1 ml EGM-2 medium without FGF2 and VEGF_165_ and resuspended in EGM-2 medium without FGF2 and VEGF_165_ at a concentration of 200 cell-coated beads/ml. The beads were supplemented with 2.5 mg/ml of fibrinogen and 0.15 units/ml of aprotinin (both from Sigma-Aldrich). Fifty microliters of fibrinogen/bead solution was added to 0.0625 units of thrombin (Sigma-Aldrich) in one well of a 96-well tissue culture plate. The fibrinogen/bead solution was allowed to clot for 5 min at room temperature and then transferred to cell culture incubator for 30 min. EBM-2 medium (150 µl) with either FGF2 (R&D Systems) or VEGF_165_ (R&D Systems) both at 20 ng/ml containing different oligosaccharides at 50 µg/ml (equivalent molar concentrations are shown in Table 1), PD173074 at 0.5 µM or SU4312 at 1 µM concentration was added and allowed to equilibrate for 3 hours. Normal human dermal fibroblasts (NHDFs) were layered on top of the clot at 0.4×10^4^ cells/well. Medium was changed after 3 days and after a further 4 days tubules were stained with Calcein-AM (1 µg/ml; Invitrogen) for 1 hour before counting tubules on each bead. Tubules were visualized on a standard inverted microscope at ×10 magnification (Solent Scientific).

### Aortic ring assay

A 96 well plate was coated with 50 µl fibrinogen solution made up in serum-free DMEM as described in the tube assay and left to set at 37°C. Thoracic aortas were removed from mice that had undergone cardiac puncture to remove blood and sacrificed by cervical dislocation. Aortas were transferred into ice-cold serum-free DMEM. The peri-aortic fibroadipose tissue was removed, aortas sectioned into 1 mm long aortic rings which were washed extensively in DMEM. Aortic sections were placed on top of fibrin gels and overlaid with 75 µl of fibrinogen gel which was allowed to set for 30 min before adding 150 µl of EBM-2 medium supplemented with 0.1% FBS and either FGF2 or VEGF_165_ plus or minus 12-mer 2SNS oligosaccharide at 50 µg/ml (14.7 µM) concentration. FGFR inhibitor PD173074 (0.5 µM) and VEGFR2 inhibitor SU4312 (1 µM) were added 1 hour prior to the addition of FGF2 or VEGF_165_, respectively. The medium was refreshed after 3 days. Aortas were stained either with Calcein-AM (1 µg/ml; Invitrogen) or with FITC-conjugated isolectin B4 (2 µg/ml; Sigma). Tubes were visualized by fluorescence microscopy at ×5 magnification (Solent Scientific). For quantification of aortic tube network phase contrast images were taken at ×4 magnification on the Zeiss Axiovert 200 M (Zeiss) enclosed in a full environmental chamber. Metamorph software was used to analyse the tubular network. The total length (µm) of the tubes was evaluated for each treatment.

### Immunoblotting

HUVECs were plated in 6 well plates at 1×10^5^ cells/well in EBM-2 medium lacking SingleQuots growth supplements and containing 0.1% FBS. After 24 hours cells were dosed with FGF2 or VEGF_165_ with or without oligosaccharides at 0.1, 1, 10 and 50 µg/ml (corresponding molar concentrations are shown in Table 1), PD173074 at 0.5 µM or SU4312 at 1 µM concentration. Five or 10 min after stimulation cell lysates were prepared in Cell Lysis Buffer (Cell Signaling Technology, MA, USA) supplemented with sodium orthovanadate (1 mM), PMSF (1 mM), phosphatase inhibitor cocktail 1 (Sigma-Aldrich, 1∶100) and phosphatase inhibitor cocktail 2 (Sigma-Aldrich, 1∶100). Ten micrograms of protein were separated by SDS polyacrylamide gel electrophoresis and blotted to polyvinylidene fluoride (PVDF) membrane. Membranes were blocked with 10% non-fat dried milk in PBS supplemented with Tween-20 (PBST) for 1 hour, followed by the incubation with the primary antibodies overnight at +4°C. The following primary antibodies were used: anti-phospho-ERK1/2 (pThr202/Tyr204; clone E10, #5120) diluted 1∶2000, rabbit polyclonal anti-ERK1/2 (#9102) at 1∶500, rabbit polyclonal anti-phospho-FRS2α (pTyr196; #3864) at 1∶500, rabbit monoclonal anti-phospho-AKT (pSer473; clone 193H12, #4058) at 1∶500, rabbit polyclonal anti-AKT (#9272) at 1∶500, rabbit monoclonal anti-phospho-VEGFR-2 (pTyr1175; clone 19A10, #2478) at 1∶500, rabbit monoclonal anti-VEGFR-2 (clone 55B11, #2479) at 1∶500 (all from Cell Signaling Technology), rabbit polyclonal anti-phospho-VEGFR-2 (pTyr1214; Invitrogen, #441052) at 1∶500 and anti-GAPDH (Abcam, #ab9485) at 1∶2000. The membranes were washed with PBST and incubated in horseradish peroxidase-conjugated goat anti-rabbit or anti-mouse IgG (Sigma-Aldrich) diluted 1∶2000 in 5% nonfat dried milk in PBST. Bound secondary antibody was detected by chemiluminescence (PerkinElmer, MA, USA). Densitometry analysis was applied to each blot to quantify the intensity of bands, followed by normalization against loading controls. Average fold-change as compared to the stimulation with the growth factor alone was derived from three independent experiments.

### Growth factor binding to endothelial cell surface

Binding of FGF2 or VEGF to the human umbilical vein endothelial (HUVE) cell surface was determined by using FGF2 and VEGF biotinylated fluorokine kits (R&D Systems, #NFFB0 and NFVE0, respectively) and following manufacturer's instructions. Briefly, HUVECs were collected using cell dissociation buffer (Invitrogen), centrifuged at 500 g for 5 min and then washed twice in PBS. Ten ng of biotinylated FGF2 or VEGF with or without oligosaccharides or specific blocking antibodies were incubated at 37°C for 1 hour prior to mixing with HUVECs for 1 hour at 4°C. Avidin-FITC was added for 30 min at 4°C in the dark. Cells were washed twice in 2 ml of cell wash RDF1 buffer, resuspended in 0.2 ml of RDF1 buffer and the binding of growth factors to the cell surface was performed using FacsCalibur (BD Biosciences, CA, USA). Data was analysed by using FlowJo Analytical software (Tree Star Inc., Ashland, USA).

### Statistical analyses

Data are expressed as the mean ±SE. For comparison of groups, the Student's *t* test was used. A level of *P* <0.05 was considered as statistically significant.

## Results

### Chemical synthesis of oligosaccharides

**Figure 1:**
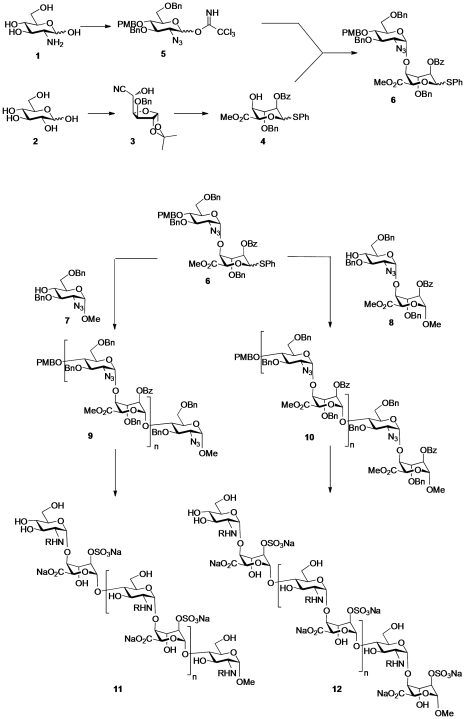
Strategy for assembly of odd- and even-numbered oligosaccharides. D-Glucose was converted to the L-ido cyanohydrin **3** and further modifications gave the L-iduronic acid acceptor **4**. This acceptor was coupled with the donor **5**, derived from D-glucosamine **1**, to yield the disaccharide donor **6** containing a reactive thioglycoside unit. The donor disaccharide **6** was elongated by reaction with either acceptor monoglucoside **7** or with acceptor disaccharide **8**. The resulting tri- and tetrasaccharides **9** and **10**, were further homologated by iterative cycles consisting of a 2-step process of selective removal of PMB group followed by addition of another disaccharide donor unit, **6**. The final odd- and even-numbered oligomers **9** and **10**, respectively, were then deprotected by sequential ester hydrolysis to allow 2-*O*-sulfation, followed by hydrogenolysis to remove benzylic ethers and reduce azido groups and finally an optional selective *N*-sulfation to yield the oligomers **11** and **12**. **11**: 7-mer 2S: n = 2, R = -H; 7-mer 2SNS: n = 2, R = -SO_3_Na; 9-mer 2S: n = 3, R = -H; and 9-mer 2SNS: n = 3, R = -SO_3_Na. **12**: 8-mer 2S: n = 2, R = -H; 8-mer 2SNS: n = 2, R = -SO_3_Na; 10-mer 2S: n = 3, R = -H; 10-mer 2SNS: n = 3, R = -SO_3_Na; 12-mer 2S: n = 4, R = -H; and 12-mer 2SNS: n = 4, R = -SO_3_Na.

We previously described an iterative synthesis of HS oligosaccharides with variable length and sulfation patterns [14]. Oligosaccharides comprising 7 to 12 saccharide residues were assembled from disaccharide precursors bearing protective groups (Figure 1). To generate the requisite α-D-glucosamine-(1→4)-α-L-iduronic acid disaccharide units (**6** and **8**), D-glucosamine **1** was converted into glucoazide donor derivative **5** in 8 chemical steps and D-glucose was converted into L-iduronic acid acceptor **4**, *via* L-ido cyanohydrin **3**, also in 8 steps [14], [15]. Both monosaccharides contain orthogonal organic protecting groups, namely carboxylic ester groups (Bz: benzoyl) and benzylic ethers (PMB: *p*-methoxybenzyl and Bn: benzyl). Compounds **4** and **5** were coupled together using the Schmidt trichloroacetimidate method to give disaccharide **6**. Further elongation using this disaccharide donor was achieved towards either even- or odd-numbered oligosaccharides by activation of the phenylthioglycoside donor unit in **6** by *N*-iodosuccinimide (NIS) for reaction with an appropriate acceptor saccharide. For synthesis of odd-numbered saccharides, **6** was reacted with the methyl glycoside (reducing end capped) monosaccharide acceptor derivative **7** to provide the intermediate trisaccharide **9** (n = 1). For synthesis of even-numbered oligosaccharides, donor disaccharide **6** was reacted with disaccharide acceptor **8**, yielding the intermediate tetrasaccharide **10** (n = 1). The odd- and even-numbered sequences **9** and **10** were extended from 3-mer up to 9-mer and from 4-mer up to 12-mer, respectively, by iterative cycles of PMB removal with cerium ammonium nitrate (CAN), unmasking a new *O*-4 acceptor hydroxyl, followed by iterative NIS-activated glycosylation with disaccharide donor **6**. The final protected oligosaccharides were then elaborated into the target sulfated HS sequences. This was achieved by sequential removal of ester moieties by basic hydrolysis and sulfation with pyridine sulfur trioxide complex, effecting selective 2-*O*-sulfation. Subsequent palladium-catalysed hydrogenolysis removed the benzylic ether protecting groups and reduced the 2-azido groups. Finally, an optional selective *N*-sulfation using pyridine sulfur trioxide complex in water yielded **11** and **12** (R = H or sulfate) as single chemical entities. This strategy thus provided two homogeneously sulfated sequences of defined length containing sulfates at either the 2-*O* position of iduronate (2S) or at both the 2-*O*-position of iduronate and *N*-position of glucosamine (2SNS; Table 1).

To confirm the structure of synthetic oligosaccharides we performed mass spectrometry analysis (Supporting Information Methods S1) using fully protected compounds, since sulfated heparin-like oligosaccharides are notoriously difficult to ionise due to their high acidity and charge. We found that the mass spectrometry of the protected intermediate oligosaccharides **9** and **10** (7-mer to 12-mer) yielded good spectra when a dithranol matrix was used (Figures S1, S2, S3, S4 and S5). The [M+Na^+^] positive ion was observed, although in some oligosaccharides, namely 10-mer and 12-mer, a [M+126+Na^+^] peak was significant (Figures S4 and S5). This peak relates to an iodine atom linked to the PMB group; an impurity formed during the coupling of oligosaccharides. The impurity could not be separated from the expected product, but in the later steps of synthesis both the PMB and iodo-PMB groups were removed giving the same final products.

To confirm sulfation pattern in 2SNS oligosaccharides we used heparinases to degrade these compounds to component disaccharides which were then analysed by strong anion exchange high-performance liquid chromatography (Supporting Information Methods S1; Figure S6). The majority of disaccharides which were present in 2SNS oligosaccharides were disulfated UA(2S)-GlcNS disaccharides as determined by comparison with the elution times of HS standards (Figure S6; Table S1). However, a low amount of monosulfated UA-GlcNS disaccharides were also present in 8-mer, 9-mer and 12-mer oligosaccharides (Figure S6; Table S1). Enzymatic digest of all oligosaccharides was incomplete yielding approximately 9–22% of unpolymerized tetrasaccharides (Figure S6; peak 4); a common limitation of enzymatic cleavage of HS. Size exclusion chromatography confirmed that the material eluting off the SAX column at 30–35 minutes is a mixture of sulfated tetrasaccharides (data not shown).

### Synthetic oligosaccharide composition determines binding affinity to FGF2 and VEGF

**Figure 2:**
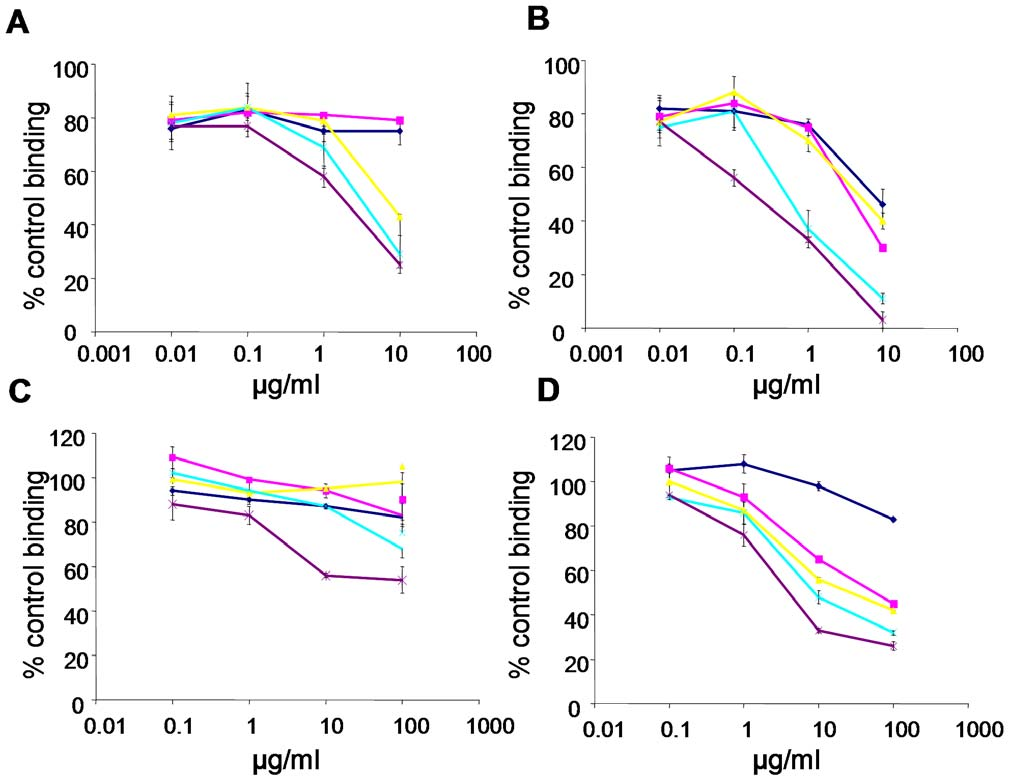
Synthetic oligosaccharides compete with HS for binding to angiogenic growth factors. (**A–B**) FGF2 and (**C–D**) VEGF_165_ binding to immobilized HS in the presence of increasing concentrations of 2S species (**A** and **C**) and 2SNS species (**B** and **D**) oligosaccharides. Data is expressed as a percentage of HS binding in the absence of the competing oligosaccharide. The mean ± SD (n = 4) is shown. Dark blue lines - 7-mers, magenta lines – 8-mers, yellow lines – 9-mers, turquoise lines – 10-mers and purple lines - 12-mers.

To determine whether the synthetic oligosaccharides bind to specific angiogenic cytokines, such as FGF2 and VEGF_165_, we evaluated the ability of oligosaccharides to inhibit cytokine binding to HS immobilized on GAG binding plates. FGF2 binding to HS was unaffected by 2S 7- and 8-mer oligosaccharides, whereas further increase in length allowed oligosaccharides to compete with HS, such that the 2S 12-mer reduced FGF2 binding to HS by 75% at 10 µg/ml (3.6 µM) concentration (Figure 2A). The 2SNS species more potently inhibited FGF2 binding to HS (Figure 2B). The 2SNS 7-mer, 8-mer and 9-mer oligosaccharides decreased binding to HS by 46%, 30% and 40%, respectively, while 10-mer and 12-mer oligosaccharides inhibited FGF2 binding to HS by >90% at 10 µg/ml concentration (Figure 2B; Table 1). The 2SNS 12-mer oligosaccharide was the most potent in that a concentration as low as 100 ng/ml (29 nM) was sufficient to inhibit 44% of binding to HS (Figure 2B).

Saccharides with 2-*O*-sulfates only were weak competitors for the interaction between HS and VEGF_165_ binding (Figure 2C), whereas the 2SNS oligosaccharides were more effective competitors and the length of oligosaccharide contributed to the affinity for VEGF_165_ (Figure 2D). However, the concentrations required to achieve similar degree of inhibition of VEGF_165_ binding to HS were 10-fold higher than those required for FGF2 and only 12-mer 2SNS prevented VEGF_165_ binding to HS by 67% at 10 µg/ml (2.9 µM) concentration (Figure 2D).

### Oligosaccharides are moderately potent inhibitors of FGF2- and VEGF_165_-induced endothelial cell proliferation

**Figure 3:**
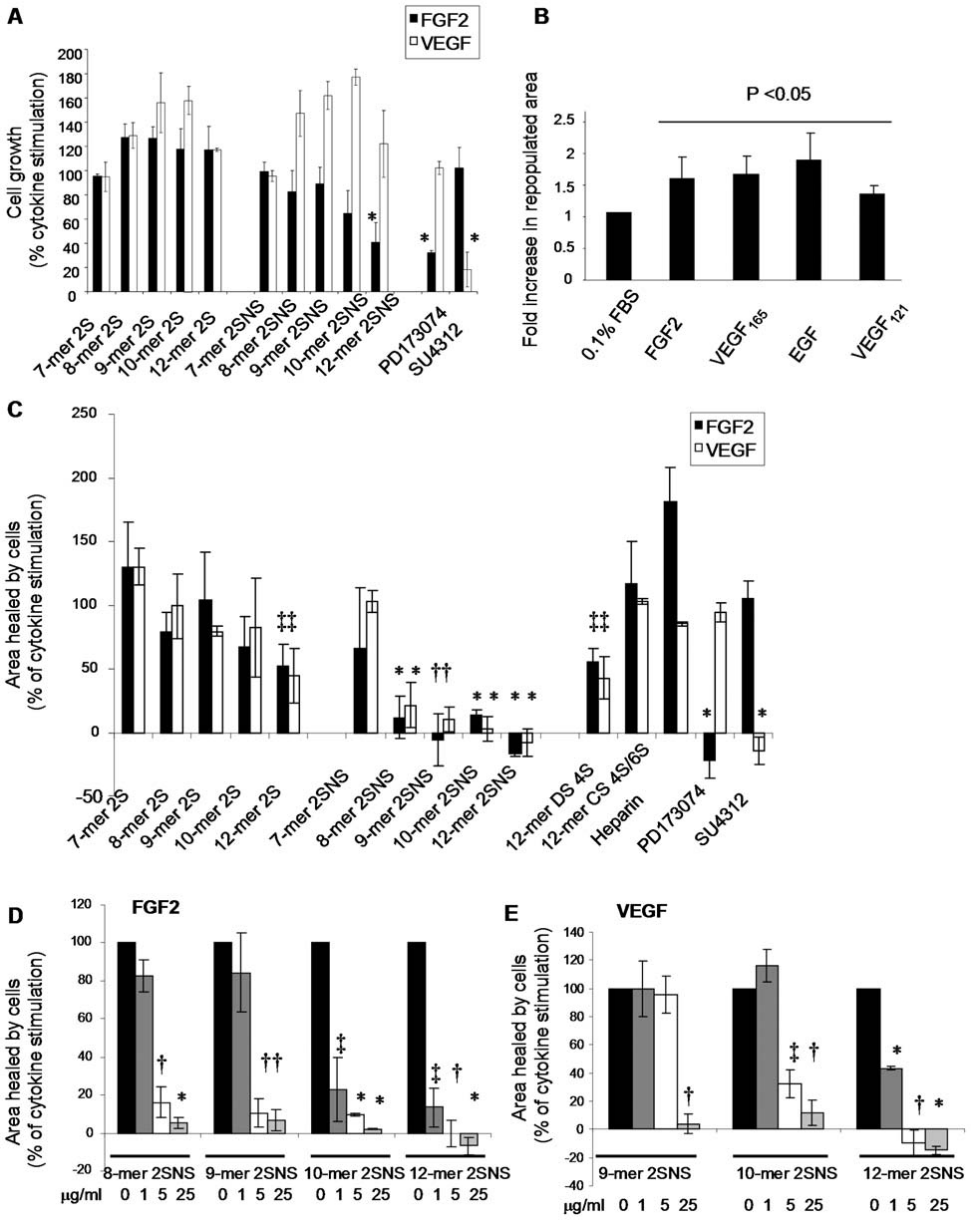
Oligosaccharides inhibit growth factor-induced endothelial cell proliferation and migration. (**A**) Effect of oligosaccharides on endothelial cell proliferation. HUVECs were incubated with or without growth factors and oligosaccharides were added at 50 µg/ml (corresponding molar dose for each oligosaccharide is shown in Table 1), PD173074 at 0.5 µM and SU4312 at 1 µM concentration. Cell proliferation after 96 hour incubation was evaluated using SRB assay. The increase in growth factor-induced cell proliferation is expressed as 100% (control). Treatment effect is shown as a percentage of a control.Results are represented as the mean ±SD (n = 3). *, *P* = 0.024. (**B**) Cytokines stimulate endothelial cell migration. Confluent layers of serum-starved immortalized HUVECs were wounded and FGF2 (20 ng/ml), VEGF_165_ (20 ng/ml), EGF (20 ng/ml) and VEGF_121_ (20 ng/ml) were added to stimulate cell migration into the wound. The wound area at baseline and after 24 h was measured. Fold increase in the repopulated area is represented as the mean ±SD (n = 3). *P*<0.05. (**C**) Effect of oligosaccharides (all at 50 µg/ml; Table 1), heparin (50 µg/ml), PD173074 (0.2 µM) or SU4312 (0.4 µM) on the cytokine-induced repopulated wound area was tested as in B. The area that healed during 24 hours in the presence of cytokines when compared to serum-starved cells is expressed as 100%. The effect of oligosaccharides, heparin and receptor tyrosine kinase inhibitors is expressed as a percentage of repopulated area induced by a cytokine alone. The mean ± SD (n = 3) is shown. *, *P*<0.0001; †, *P*<0.001; ‡, *P*<0.005. (**D–E**) Inhibition of FGF2- or VEGF_165_-induced wound closure at increasing concentrations of oligosaccharides that inhibit cell migration by more than 80% at a maximal 50 µg/ml concentration. The mean ± SD (n = 2) is shown. *, *P*<0.001; †, *P*<0.005; ‡, *P*<0.05.

During neovascularisation, rapidly proliferating endothelial cells are incorporated into new vessels [16]. Therefore, we first assessed whether oligosaccharides inhibit endothelial cell proliferation induced by FGF2 or VEGF_165_. Oligosaccharide species bearing only *O*2 sulfation had minimal effects on FGF2- or VEGF_165_-dependent cell proliferation (Figure 3A). In contrast, 10-mer 2SNS and 12-mer 2SNS oligosaccharides dosed at 50 µg/ml concentration (16.9 µM 10-mer 2SNS and 14.7 µM 12-mer 2SNS) inhibited specifically FGF2-stimulated cell proliferation by 35% and 59%, respectively (Figure 3A). A similar degree of inhibition was observed with the FGF receptor (FGFR) tyrosine kinase inhibitor PD173074 and a selective inhibitor of VEGF and PDGF receptor tyrosine kinases SU4312, albeit at lower concentrations (Figure 3A). PD173074 inhibited FGF2-induced cell proliferation by 68% at 0.5 µM concentration, while at 1 µM concentration SU4312 reduced VEGF_165_-induced cell proliferation by 81%, indicating that receptor tyrosine kinase inhibitors are more potent inhibitors of FGF2- and VEGF_165_-induced endothelial cell proliferation than 2SNS 10-mer and 12-mer oligosaccharides.

### Oligosaccharides inhibit cytokine-mediated endothelial cell migration

Endothelial cell motility and the ability to respond to angiogenic growth factor gradients are essential processes for angiogenesis [17]. Therefore, we assessed endothelial cell migration into mechanically generated wounds in response to FGF2, VEGF_165_, EGF and VEGF_121_. The latter two cytokines were studied as they are not dependent on HS for their biological activity. The area repopulated by SV40-immortalized HUVECs (EVLC2 cell line) following 24 hour stimulation with FGF2, VEGF_165_ and EGF increased by approximately 2-fold when compared to un-stimulated serum-starved cells, while VEGF_121_ induced endothelial cell migration to a lesser extent (Figure 3B). Next we tested if 2S and 2SNS species of oligosaccharides could abrogate FGF2- and VEGF_165_-induced healing of the wounded area. 2S oligosaccharides had little effect on cell migration (Figure 3C), with the exception of 12-mer 2S, which at 50 µg/ml (18 µM), reduced cytokine-induced cell advancement by 55% (Figure 3C). To compare the functional significance of oligosaccharide charge density and specific saccharide structure for 12-mers bearing one sulfate group on FGF2- or VEGF_165_-mediated cell migration, we tested the activity of dodecasaccharides composed of 4-*O* sulfated dermatan sulfate (DS 4S) and a mixture of either 4-*O* or 6-*O* sulfated chondroitin sulfate (CS 4S/6S). The DS 4S 12-mer inhibited both FGF2- and VEGF_165_-induced cell migration to the same degree as the synthetic 12-mer 2S, while CS 4S/6S 12-mer was inactive suggesting that the specificity of sugar structure contributes to the anti-migratory activity (Figure 3C). 2SNS oligosaccharide species showed strong structure-dependent anti-migratory activity (Figure 3C). The length of 2SNS oligosaccharides was crucial in selective targeting of FGF2- or VEGF_165_-induced endothelial cell repopulation (Figure 3C). 2SNS oligosaccharides containing at least 8 saccharide residues were required to inhibit FGF2-induced wound closure by 89%, while molecules containing 9 saccharide residues achieved the same degree of inhibition of VEGF_165_-dependent wound closure (Figure 3C). The 12-mer 2SNS oligosaccharide was the only compound that completely inhibited FGF2- and VEGF_165_-dependent cell migration into the wound at a concentration as low as 5 µg/ml (1.45 µM; Figure 3D-E). No effect was seen when 2SNS 8–12-mer oligosaccharides were tested on non-stimulated cells (data not shown) or cells stimulated with EGF and VEGF_121_ (Figure S7).

To determine the IC_50_ values of 2SNS oligosaccharides that inhibited cytokine-induced cell advancement, the wounds were treated with increasing concentrations of 2SNS 8-mer, 9-mer, 10-mer and 12-mer oligosaccharides together with the specific angiogenic cytokines and repopulated areas at each concentration were assessed. For FGF2 the oligosaccharide IC_50_ values ranked in the following order: 12-mer 2SNS*>*10-mer 2SNS*>*9-mer 2SNS*>*8-mer 2SNS (Table 2). The 9-mer 2SNS and 10-mer 2SNS were significantly less potent in inhibiting VEGF_165_–mediated cell advancement into the wound when compared to FGF2, while 12-mer 2SNS oligosaccharide was the most potent inhibitor that targeted FGF2 and VEGF_165_ with a similar potency (Table 2). More strikingly, 12-mer 2SNS IC_50_ values of FGF2- or VEGF_165_-mediated cell migration were similar to those of PD173074 and SU4312 compounds, respectively (Table 2).

**Table 2:** IC_50_ values of biologically active oligosaccharides and FGFR and EGFR-2 inhibitors in endothelial cell migration assay.

Oligosaccharide/RTKi	FGF2		VEGF_165_	
	µg/ml	µM	µg/ml	µM
8-mer 2SNS	2.86	1.2	ND	ND
9-mer 2SNS	2.11	0.83	9.17	3.6
10-mer 2SNS	0.30	0.1	3.54	1.2
12-mer 2SNS	0.38	0.11	0.58	0.17
PD173074	-	0.082	ND	ND
SU4312	ND	ND	-	0.19

The mean of at least two independent experiments is represented. No more than 2-fold differences between IC_50_s in two independent experiments were observed. ND - not determined; RTKi – receptor tyrosine kinase inhibitor.

### Structure-dependent anti-angiogenic activity of oligosaccharides

**Figure 4:**
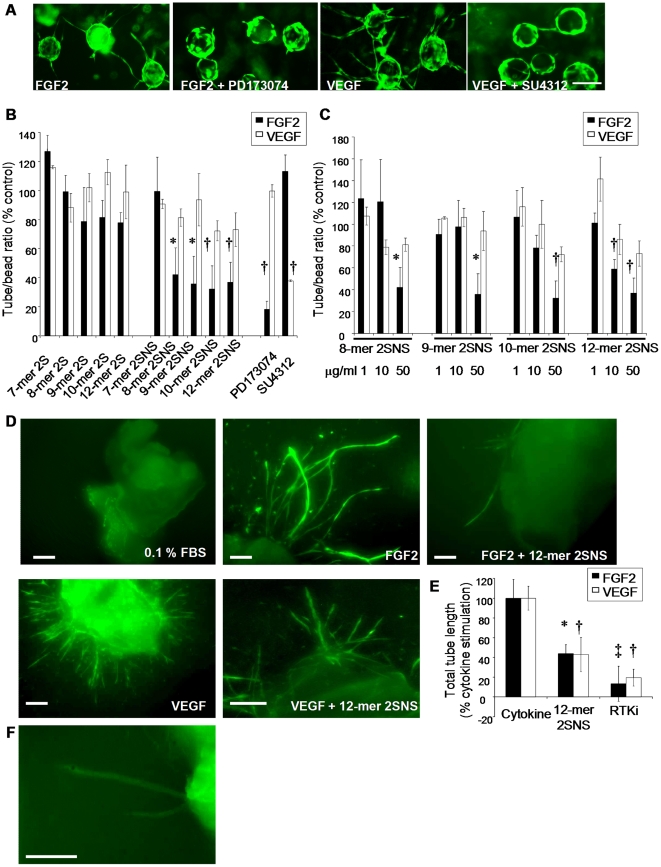
Oligosaccharides inhibit growth factor-induced endothelial tube formation. (**A**) HUVEC coated beads were embedded in fibrin gels and treated with either FGF2 or VEGF_165_ alone or with FGF2 in the presence of PD173074 (0.5 µM) and VEGF_165_ in the presence of SU4312 (1 µM) for 6 days. Tubes were visualised by staining with Calcein-AM. Scale bar, 100 µm. (**B**) HUVEC coated beads were treated with FGF2 or VEGF_165_ mixed with oligosaccharides at 50 µg/ml concentration (equivalent molar concentrations are shown in Table 1). PD173074 and SU4312 were used at 0.5 µM and 1 µM concentration, respectively. The average number of tubes per bead (20–50 beads were evaluated) was quantified. FGF2 or VEGF_165_ alone treatment represents a control (100%). Percentage in number of tubes per bead in each treatment when compared to the control is shown as mean ± SD (n = 2–4). *, *P*<0.035; †, *P*<0.02. (**C**) Oligosaccharide dose-dependent inhibition of FGF2- or VEGF_165_-induced endothelial tube formation. Response to 1, 10 and 50 µg/ml concentration (Table 1) of oligosaccharides was evaluated as in B. The mean ± SD (n = 2) is shown. *, *P*<0.035; †, *P*<0.02. (**D**) Mouse aortic rings were embedded in fibrin gels supplemented with the medium with no growth factors (0.1% FBS) or with FGF2, FGF2 plus 12-mer 2SNS (50 µg/ml; 14.7 µM), VEGF_165_ or VEGF_165_ plus 12-mer 2SNS (50 µg/ml; 14.7 µM). Tube growth was evaluated after 6 days. Aortas were stained with Calcein-AM. Scale bars, 300 µm. (**E**) Quantification of total aortic tube length was performed using MetaMorph software and expressed as a percentage of a total tube length induced by the growth factor alone. 12-mer 2SNS was used at 50 µg/ml (14.7 µM), PD173074 at 0.5 µM and SU4312 at 1 µM concentration. RTKi – receptor tyrosine kinase inhibitors. The mean ±SD (n = 3–5) is shown. *, *P* = 0.01; †, *P*≤0.003; ‡, *P*<0.0001. (**F**) Tubular structures derived from aortas show endothelial cell-specific staining with FITC-conjugated isolectin B4. Scale bar, 300 µm.

Endothelial tubulogenesis is a complex process that requires multiple endothelial cell functions, including proliferation, migration, invasion and differentiation [16]. Since we observed oligosaccharide structure-dependent inhibition of endothelial cell migration, we examined how different oligosaccharide species affect FGF2- and VEGF_165_-dependent endothelial tube formation. FGF2 or VEGF_165_ stimulation was essential for the vessel-like structures to grow from HUVEC-coated beads in fibrin gels (Figure 4A). FGFR inhibitor PD173074 and VEGFR-2 inhibitor SU4312 inhibited tube formation in response to FGF2 or VEGF_165_, respectively (Figure 4A). Similar to the effects on cell migration, the 2S oligosaccharides did not affect FGF2- or VEGF_165_-induced endothelial tube formation (Figure 4B). In contrast, the 2SNS oligosaccharides with a minimum 8 residues at 50 µg/ml concentration inhibited FGF2-dependent tube formation by 60–70% (Figure 4B; equivalent molar concentrations are shown in Table 1). 2SNS oligosaccharides were less effective inhibitors of VEGF_165_-mediated tubulogenesis. Only 2SNS 10- and 12-mers reduced the number of tubes by 28% (Figure 4B). At lower oligosaccharide concentrations (10 µg/ml; Table 1) only 12-mer 2SNS inhibited FGF2- or VEGF_165_-mediated tube formation by 42% and 16%, respectively, while shorter oligosaccharides were less potent (Figure 4C). To test 2SNS 12-mer's activity in a more physiologically relevant model of angiogenesis, we evaluated how 12-mer 2SNS affects FGF2- or VEGF_165_-induced formation of angiogenic vessel-like structures derived from mouse aortic rings embedded in fibrin gels (Figure 4D). As in the bead assay, FGF2 or VEGF_165_ significantly increased formation of aorta-derived tube structures (Figure 4D). 12-mer 2SNS oligosaccharide at 50 µg/ml (14.7 µM) concentration reduced FGF2- or VEGF_165_-dependent total tube length by 56 and 57%, respectively (Figure 4E). The endothelial nature of the tubes was confirmed by staining the aortas with FITC-conjugated isolectin B4 (Figure 4F).

### Oligosaccharides reduce FGF2 binding to endothelial cell surface

**Figure 5:**
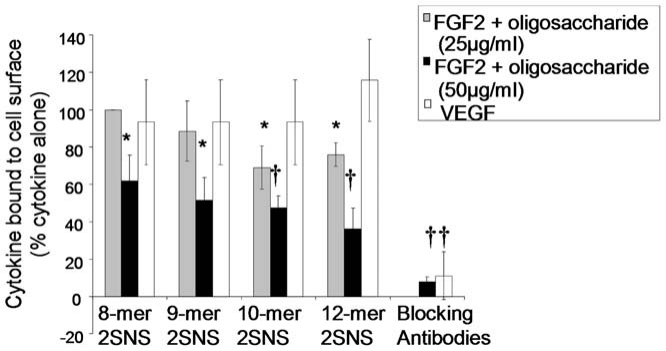
2SNS species of oligosaccharides reduce FGF2, but not VEGF_165_, binding to endothelial cell surface. HUVECs were mixed with biotin-labelled FGF2 or VEGF_165_ alone or in the presence of 2SNS oligosaccharides at either 25 µg/ml (FGF2 treatment; grey bars) or 50 µg/ml (FGF2 treatment; black bars and VEGF_165_ treatment; white bars) concentration (equivalent molar doses are shown in Table 1). The binding of growth factors to HUVE cell surface was determined by addition of avidin-FITC and analysed by FACS. FGF2 or VEGF_165_ binding in the absence of oligosaccharides or blocking antibodies is expressed as 100%. Percentage of growth factor binding when compared to the sample incubated without oligosaccharides or blocking antibodies is shown as the mean ±SD (n = 2). *, *P*<0.05; †, *P*≤0.01.

To test whether oligosaccharides that showed activity in the tube formation and migration assays affect FGF2 or VEGF_165_ binding to HUVE cell surface, we treated cells with biotinylated FGF2 or VEGF_165_ in the presence or absence of oligosaccharides and determined their levels on the cell surface. 12-mer 2SNS was the most effective oligosaccharide in reducing cell surface bound FGF2 by more than 60% at 50 µg/ml (14.7 µM) concentration (Figure 5). However, at a lower, although biologically effective, concentration (25 µg/ml) none of the oligosaccharides prevented FGF2 binding to cells (Figure 5). VEGF_165_ binding to endothelial cells was unaffected by any of the 2SNS oligosaccharides (Figure 5), suggesting that prevention of a complex formation between FGF2 or VEGF_165_ and their receptors might not be the main mechanism responsible for the inhibition of endothelial cell functions by oligosaccharides.

### The 2SNS dodecasaccharide inhibits FGF2- and VEGF_165_-induced peripheral accumulation of phosphorylated FAK and actin reorganization

**Figure 6:**
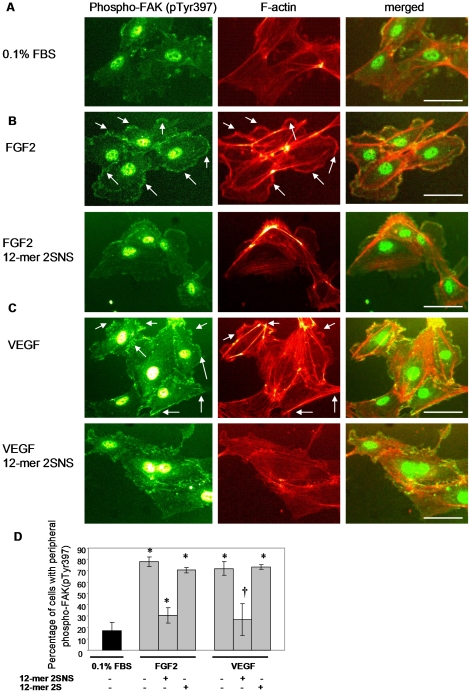
FGF2- and VEGF_165_-induced peripheral accumulation of activated FAK and F-actin is inhibited by 2SNS 12-mer oligosaccharide. (**A**) Lack of peripheral accumulation of FAK phosphorylated on tyrosine 397 and F-actin in serum-starved HUVECs. Cells were co-stained with anti-phospho-FAK (pTyr397) antibody (left image) and phalloidin-AlexaFluor568 (middle image). Merged view is represented in the right image. Scale bar, 75 µm. (**B–C**) Peripheral FAK phosphorylated at tyrosine 397 and F-actin are detected after 10 min stimulation with FGF2 (**B**, upper images) or VEGF_165_ (**C**, upper images). 12-mer 2SNS oligosaccharide prevents peripheral localization of phosphorylated FAK and F-actin in response to FGF2 (**B**, lower images) or VEGF_165_ (**C**, lower images). Merged images are presented in the right panel. Scale bars, 75 µm. (**D**) Percentage of cells with peripheral FAK phosphorylated on tyrosine 397 in unstimulated (0.1% FBS) or FGF2 or VEGF_165_ stimulated cells in the absence or presence of 12-mer 2SNS or 12-mer 2S is shown as the mean ±SD (n = 3). *, *P*<0.0005; †, *P*<0.01.

The above data demonstrate that the 2SNS species inhibit cytokine induced endothelial cell migration and tube formation. We therefore investigated the mechanism for this observation. Actin remodelling into lamellipodia, filopodia and stress fibers is an essential cell response component required for cytokine-induced cell migration [17]. Migrating cells assemble dynamic focal adhesions at the leading edge in a process that crucially depends on the spatial activation/inactivation cycles of focal adhesion kinase (FAK) [17], [18]. With this in mind, we first tested how FGF2 and VEGF_165_ affect FAK phosphorylation on tyrosines 397 and 861 which is required for FAK activation [18], as well as the organization of F-actin in stimulated cells. Serum-starved HUVECs were stimulated with FGF2 or VEGF_165_ and co-stained with the antibody recognizing FAK phosphorylated at tyrosine 397 or 861 and phalloidin-AlexaFluor568 to visualize F-actin. Peripheral accumulation of phosphorylated FAK and F-actin were detected only in FGF2- and VEGF_165_-stimulated (Figure 6B-C, upper panels), but not in serum-starved cells (Figure 6A). Localization pattern of FAK phosphorylated on tyrosine 861 was identical to phospho-FAK (pTyr397) (data not shown). Treatment with the 12-mer 2SNS oligosaccharide completely abrogated FGF2- and VEGF_165_-induced accumulation of peripheral FAK phosphorylated at tyrosine 397 (Figure 6B-C, lower left panels) or 861 (data not shown) and actin-rich lamellipodia-like structures (Figure 6B-C, lower middle panels). Quantification of cells with peripheral phosphorylated FAK following each treatment showed that 12-mer 2SNS oligosaccharide, but not 12-mer 2S, significantly prevented peripheral activation of FAK in response to FGF2 or VEGF_165_ treatment (Figure 6D).

### Oligosaccharides inhibit FGF2- and VEGF_165_-induced receptor activation and signalling

**Figure 7:**
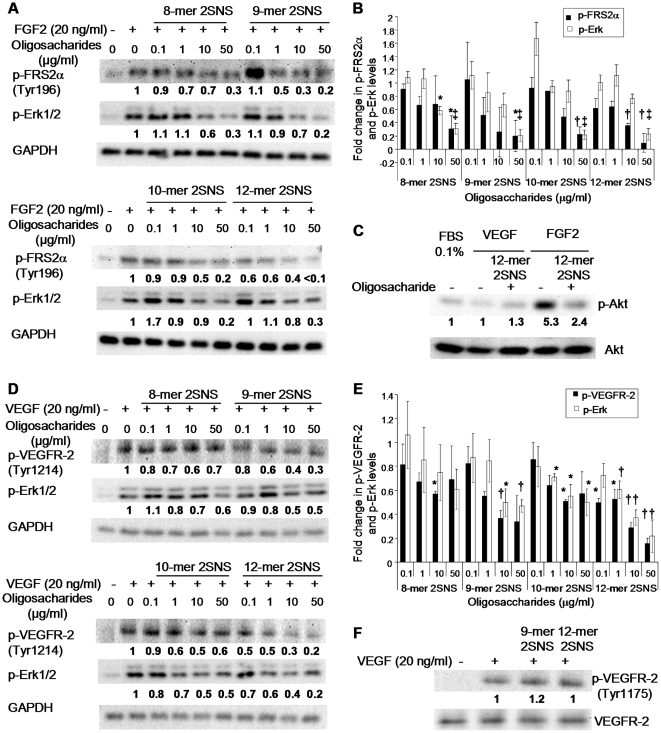
Oligosaccharides inhibit FGF2- and VEGF_165_-induced receptor activation and signalling. (**A**) Serum-starved HUVECs were stimulated with FGF2 for 10 min in the absence or presence of increasing concentrations of indicated oligosaccharides. Phosphorylated FRS2 and Erk were detected by Western blotting. Equal protein loading was monitored by probing with the anti-GAPDH antibody. The values below each blot represent an average normalized fold change in the intensities of bands as compared to the bands corresponding to FGF2 stimulated cells in the absence of oligosaccharides. The normalized fold change was derived from three independent experiments. (**B**) Fold change of phosphorylated FRS2 and Erk levels in FGF2 treated cells in the presence of oligosaccharides as compared to the FGF2 stimulated cells without oligosaccharide treatment. Amount of phosphorylated FRS2 and Erk as determined by densitometric evaluation was normalized against corresponding GAPDH levels. Values are the mean ± SEM (n = 3). *, *P*<0.05; †, *P*<0.01; ‡, *P*<0.0001. (**C**) Biologically active 12-mer 2SNS inhibits FGF2-induced activation of PI3K/Akt pathway. Serum-starved HUVECs were exposed to FGF2 (20 ng/ml) or VEGF_165_ (20 ng/ml) for 10 min in the absence or presence of 12-mer 2SNS (50 µg/ml; 14.7 µM). Phospho-Akt (Ser473) was detected by immunoblotting with the anti-phospho-Akt antibody recognising Akt phosphorylation on serine 473. Total Akt protein levels were assessed by probing with the anti-Akt antibody. Average fold change values derived from three independent experiments show no stimulation of Akt phosphorylation by VEGF_165_ and inhibition of FGF2-induced phosphorylation of Akt by 12-mer 2SNS. (**D**) VEGF_165_ stimulation was performed in the absence or presence of increasing concentrations of respective oligosaccharides. Phosphorylation of VEGFR-2 on tyrosine 1214 and phospho-Erk were detected by immunoblotting with the respective antibodies as shown. GAPDH levels show the total protein levels. Average fold change derived from three independent experiments shows significant activity of 12-mer 2SNS in inhibiting phosphorylation of VEGFR-2 and Erk. (**E**) Fold change of phosphorylated VEGFR-2 (Tyr1214) and Erk levels in VEGF_165_ stimulated cells in the presence of oligosaccharides as compared to VEGF_165_ stimulated cells without oligosaccharide treatment. The amount of phosphorylated proteins was normalized against corresponding GAPDH levels. Values are the mean ± SEM (n = 3). *, *P*<0.05; † *P*<0.01. (**F**) 12-mer 2SNS is not affecting phosphorylation of VEGFR-2 on tyrosine 1175. VEGF_165_ treatment was performed for 5 min in the absence or presence of 9-mer 2SNS or 12-mer 2SNS dosed at 50 µg/ml (9-mer 2SNS 19.6 µM and 12-mer 2SNS 14.7 µM) concentration. Phosphorylation of VEGFR-2 on tyrosine 1175 was detected by probing with the indicated antibody. Anti-VEGFR-2 antibody was used to determine the total VEGFR-2 protein levels. Densitometric quantification of three independent blots shows no effect on phospho-VEGFR-2 (Tyr 1175) levels.

To investigate the effect of oligosaccharides on FGF2- or VEGF_165_- induced signalling events, we investigated changes in the phosphorylation state of FGFR docking protein FRS2, VEGFR-2, Erk and Akt. The average normalized fold-change compared to the positive control was derived from three independent experiments and is shown below each blot. The phosphorylation level of FRS2 increased following stimulation with FGF2 (Figure 7A). At the maximum concentration (50 µg/ml; Table 1) 2SNS oligosaccharides containing between 8 and 12 saccharide residues significantly reduced FRS2 phosphorylation, with 12-mer 2SNS reducing FRS2 phosphorylation to the basal level (Figure 7A-B). At 0.1 µg/ml (29 nM) concentration only 12-mer 2SNS significantly diminished FRS2 phosphorylation levels, in keeping with the potency of this oligosaccharide (Figure 7A-B). Consistently, reduction of FGF2-induced Erk phosphorylation by oligosaccharides correlated with the phosphorylation status of FRS2 at a maximal 50 µg/ml concentration (Figure 7A-B). Moreover, 12-mer 2SNS oligosaccharide reduced FGF2-induced phosphorylation of Akt by almost half (Figure 7C).

Next we examined the phosphorylation of VEGFR-2 on tyrosine 1214, which has been previously implicated in the activation of Cdc42 and SAPK2/p38-mediated actin reorganization [19]. Increase in the phosphorylation of this tyrosine residue was prominent following VEGF_165_ stimulation, whereas it decreased when cells were treated with VEGF_165_ and the 12-mer 2SNS at a concentration as low as 0.1 µg/ml (29 nM) as shown by the average fold-change derived from three independent experiments (Figure 7D-E). 2SNS oligosaccharides composed of 9 and 10 saccharide residues impacted on tyrosine 1214 phosphorylation levels less significantly (Figure 7D-E). 12-mer 2SNS was the most potent oligosaccharide in reducing VEGF_165_-induced phosphorylation of Erk (Figure 7D-E). Since phosphorylation of VEGFR-2 on tyrosine 1175 is required for endothelial cell proliferation [20], we investigated whether this phosphorylation site was affected by treatment with 12-mer 2SNS and the less biologically active 9-mer 2SNS. Although VEGF_165_ prominently stimulated phosphorylation on tyrosine 1175, neither oligosaccharide reduced the levels of phosphorylation at maximal 50 µg/ml concentration (Figure 7F; Table 1), suggesting that 2SNS dodecasaccharide exerts differential inhibitory effects on VEGF_165_-regulated endothelial cell responses through the reduced phosphorylation of specific tyrosine residues in VEGFR-2.

## Discussion

In this study we present for the first time two series of synthetic, structurally defined oligosaccharides and investigate their activity in a number of cell-based assays designed to test endothelial cell proliferation, migration and tube formation in response to various angiogenic cytokines. So far organic syntheses have been reported for hexasaccharides [21], octasaccharides [22] and more recently for a dodecasaccharide [23]. However, no comprehensive biological activity data relating to oligosaccharide length and specific sulfation patterns have been described.

It is generally accepted that FGF2 can bind relatively short oligosaccharides consisting of at least four to six saccharide residues that contain iduronate 2-*O*-sulfate and glucosamine *N*-sulfate [24]–[25]. A 6-*O*-sulfate moiety is not critical for the HS-FGF2 interaction, although sulfation at this site is required for the formation of the tri-molecular signalling complex involving FGF2, HS and FGFR [5]. Our competitive binding assays show that increasing 2-*O*-sulfated oligosaccharide length to 9 saccharide residues allows 2S oligosaccharides to compete with HS for FGF2 binding (Figure 2A). This affinity is not further increased by adding extra saccharide residues to the chain, suggesting that there might be a critical length or overall charge required for FGF2 binding. Our results also show that *N*-sulfation in the presence of 2-*O*-sulfate groups significantly improves binding affinity to FGF2, while it is a prerequisite for the binding to VEGF_165_. 2SNS oligosaccharides were less potent inhibitors of the interaction between VEGF_165_ and HS than the interaction between FGF2 and HS. This reflects the fact that different HS structures are required to facilitate binding to VEGF_165_. For example, VEGF_165_ binds HS through two highly sulfated S-domains flanked by transitional regions [8] indicating that VEGF_165_ perhaps require much longer species of oligosaccharides for their optimal binding affinity.

Our study demonstrates HS fragment structure-dependent inhibition of endothelial cell functions; mainly inhibiting migration and tube formation. The level of inhibition correlated with the degree of affinity of oligosaccharides to FGF2 and VEGF_165_. Compounds containing as few as 8 saccharide residues with 2-*O*- and *N*-sulfation impacted adversely on FGF-2-induced cell migration and tube formation, while longer 2SNS oligosaccharides were active against VEGF_165_-mediated cellular responses, mainly cell migration. Similarly to our work, Leali et al. investigated the requirement for specific sulfation patterns in *Escherichia coli* K5 polysaccharide, confirming the essential requirement for *N*-sulfation for anti-angiogenic activity [26]. Moreover, sulfation in the 2-*O*- and/or 3-*O*-position in GlcA residue was also required to inhibit endothelial cell sprouting, morphogenesis and vascularisation of chick embryo chorioallantoic membrane [26]. Our studies suggest that the biological activity of oligosaccharides might also depend on the saccharides present in heparan sulfate, namely iduronate, since the DS 4S 12-mer, but not CS 4S/6S 12-mer, impacted on growth factor-induced endothelial cell migration. Since heparin activated FGF2-dependent endothelial cell migration in our assay (Figure 3C), the specificity of sulfation in an HS chain is likely to be an important determinant of the oligosaccharide's potential to support or inhibit growth factor activity.

One of the mechanisms of inhibition by oligosaccharides might involve competition for cell surface HS and therefore reduced formation of cytokine/HS/receptor signalling complexes. We saw relatively low oligosaccharide structure-dependent reduction of the endothelial cell surface-bound FGF2, whereas binding of VEGF_165_ was unaffected (Figure 5), suggesting that the mechanism of inhibition might involve the formation of non-functional signalling complexes involving FGF2 or VEGF_165_, HS and the respective receptor on the cell surface. Indeed, one recent study showed that 2SNS HS dodecasaccharides specifically form a complex with FGF2 and FGFR1c [27].

Oligosaccharides were weak inhibitors of cell proliferation, with the exception of the 12-mer 2SNS which moderately inhibited FGF2-stimulated endothelial cell proliferation. The same level of mitogenic inhibition was also observed in Ba/F3 cells expressing exogenous FGFR1 when cells were treated with HS-derived dodecasaccharide rich in 2-*O*- and *N*-sulfation [7], suggesting that this HS structure is able to prevent optimal activation of FGF-FGFR complex. Similarly, anti-angiogenic K5 polysaccharide derivative was a poor inhibitor of FGF2-induced endothelial cell proliferation [26]. How do oligosaccharides inhibit cell migration and tube formation while having only a modest effect on cell proliferation? One possibility is that oligosaccharides interfere with distinct signalling pathways that convey either proliferative or migratory signals. Despite the complexity of FGF-induced signalling, several publications suggest that the migratory activity of FGF2 requires MAP kinase pathway involving either Erk or p38 MAP kinase [28]. In our study, the most potent 12-mer 2SNS oligosaccharide significantly reduced FGF2-induced Erk phosphorylation (Figure 7A-B). Consistent with the anti-migratory function, 12-mer 2SNS oligosaccharide also abolished FGF2-induced polymerization of actin in lamellipodia-like structures rich in activated FAK (Figure 6B). Erk inhibition might also explain the inability of FGF2 to induce peripheral focal complexes via Erk substrates, such as FAK, MLCK or calpain, required for focal adhesion dynamics and cell migration [29].

We found that the most biologically active 12-mer 2SNS targeted specific VEGF_165_-induced phosphorylation sites in VEGFR-2, such that phosphorylation on tyrosine 1214 was reduced, but tyrosine 1175 phosphorylation was unaffected (Figure 7D and 7F), implying that the VEGF_165_/VEGFR-2/12-mer 2SNS complex has altered biological function. Tyrosine 1175, when phosphorylated, recruits and activates PLCγ1 which leads to the activation of protein kinase C (PKC) and cell proliferation [20]. This is consistent with our study which showed that 2SNS 12-mer oligosaccharide had a moderate effect on endothelial cell proliferation (Figure 3A). On the contrary, phosphorylation on VEGFR-2 tyrosine 1214 is required for the activation of Cdc42 and p38, actin reorganization and cell migration [19]. Another VEGF_165_-induced pathway specifically required for endothelial cell migration, but not proliferation, is activation of PLCβ3 leading to Cdc42 activation which can be sustained at lamellipodia structures [30]. In agreement, we found that 12-mer 2SNS completely abolished phosphorylation of PLCβ3 (data not shown) and accumulation of activated peripheral FAK and F-actin in response to VEGF_165_ (Figure 6C). While these data point to specific phosphorylation patterns of VEGFR-2 in response to oligosaccharides, 12-mer 2SNS could also interfere directly with VEGFR-2 associated proteins. For example, it is known that neuropilin-1 (NRP-1) and VEGFR-2 interaction is enhanced by HS and leads to increased chemotaxis in response to VEGF_165_ [31]. 12-mer 2SNS could also impinge on the ability of VEGF_165_ to activate Src and prevent the assembly of peripheral FAK phosphorylated on tyrosine 861 and integrin αvβ5 complexes, which are critical in endothelial cell adhesion and migration [32].

In conclusion, novel synthetic chemistry [14], [15] has enabled us to produce two species of HS oligosaccharides that have specific length and sulfation patterns. Using these reagents we have defined critical structural features required for the inhibition of specific cytokine-dependent endothelial cell responses. Heparin-derived oligosaccharides have been shown to be promising compounds capable of reducing physiological and pathological angiogenesis [13]. The most biologically potent inhibitory species, 2SNS dodecasaccharides, therefore presents an opportunity to develop a molecule that targets a broad spectrum of angiogenic cytokines that signal through tyrosine kinase receptors, but which have a mandatory dependence on HS for their biological activity.

## Supporting Information

Methods S1 (0.03 MB DOC)

Figure S1 Full scan mass spectrum of protected intermediate 7-mer oligosaccharide. MALDI-TOF m/z calculated for [M+Na+] (C152H156N12O39Na+): 2797 (100.0%), 2798 (90.2%), 2796 (58.1%); found: 2799.
(0.29 MB TIF)

Figure S2 Full scan mass spectrum of protected intermediate 8-mer oligosaccharide. MALDI-TOF m/z calculated for [M+Na+] (C173H176N12O46Na+): 3182 (100.0%), 3181 (98.1%), 3183 (70.6%); found: 3184.
(0.28 MB TIF)

Figure S3 Full scan mass spectrum of protected intermediate 9-mer oligosaccharide. MALDI-TOF m/z calculated for [M+Na+] (C193H197N15O50Na+): 3549 (100.0%), 3548 (88.2%), 3550 (78.2%); found: 3548.
(0.31 MB TIF)

Figure S4 Full scan mass spectrum of protected intermediate 10-mer oligosaccharide. MALDI TOF m/z calculated for [M+Na+] (C214H217N15O57Na+): 3933 (100.0%), 3934 (84.3%), 3932 (81.4%); found: 3934. m/z calculated for [M-H+I+Na+] (C214H216IN15O57Na+): 4059 (100.0%), 4060 (84.3%), 4058 (81.4%); found: 4060.
(0.35 MB TIF)

Figure S5 Full scan mass spectrum of protected intermediate 12-mer oligosaccharide. MALDI TOF: m/z calculated for [M+Na+] (C255H258N18O68Na+): 4685 (100.0%), 4686 (99.5%), 4687 (75.4%); found: 4688. m/z calculated for [M-H+I+Na+] (C255H257IN18O68Na+): 4811 (100.0%), 4812 (99.5%), 4813 (75.4%); found: 4814.
(0.38 MB TIF)

Figure S6 Disaccharide analysis of 8-mer 2SNS, 9-mer 2SNS, 10-mer 2SNS and 12-mer 2SNS oligosaccharides. Separation of disaccharides by SAX-HPLC is shown. Arrowheads show elution positions for UA-GlcNS (1), UA(2S)-GlcNAc (2), UA(2S)-GlcNS (3) and tetrasaccharides (4) as determined by comparison with elution times of HS standards. UA - uronic acid; GlcNAc - N-acetylated glucosamine; GlcNS - N-sulfated glucosamine; 2S - 2-O sulfate; E - enzymes.
(0.25 MB TIF)

Figure S7 Biologically active 2SNS oligosaccharides that inhibit FGF2-induced cell migration have no effect on EGF- and VEGF121-stimulated cell advancement. Confluent layers of serum-starved immortalized HUVECs were wounded, EGF (20 ng/ml) or VEGF121 (20 ng/ml) were added to stimulate cell migration into the wound in the absence or presence of 8-mer 2SNS, 9-mer 2SNS, 10-mer 2SNS and 12-mer 2SNS oligosaccharides dosed at 50 mg/ml concentration. The wound area at baseline and after 24 hours was measured. The area that healed in the presence of cytokines alone when compared to serum-starved cells is expressed as 100%. The effect of oligosaccharides is expressed as percentage of repopulated area by cells stimulated with the cytokine alone. Data is presented as the mean ± SD (n = 3).
(0.09 MB TIF)

Table S1 Disaccharide composition of 2SNS oligosaccharides.
(0.03 MB DOC)
