# Supplementary material for: Synthetic Heparan Sulfate Oligosaccharides Inhibit Endothelial Cell Functions Essential for Angiogenesis
Source: PLoS One. 2010 Jul 21;5(7):e11644. doi: 10.1371/journal.pone.0011644 (PMC2908126; doi:10.1371/journal.pone.0011644)
Supplement: Methods S1 — (0.03 MB DOC) [file pone.0011644.s001.doc]

**Supporting Information Methods S1**

**Mass Spectrometry**

Mass spectra of protected intermediate 7-mer, 8-mer, 9-mer, 10-mer and 12-mer oligosaccharides were obtained using a Shimadzu AXIMA Confidence MALDI time-of-flight (TOF) confidence instrument (Shimadzu Corporation, Milton Keynes, UK). The data acquisition was done using Shimadzu Biotech software. Spectra were obtained in a positive ion mode using dithranol as a matrix.

**Enzymatic depolymerisation of oligosaccharides**

Fifty μg of oligosaccharides were digested to component disaccharides using a mix of heparinase I, II and III (5 mIU of each heparinase) in 50 μL of 0.1 M sodium acetate and 0.1 mM calcium acetate (pH 7.0) at 37^o^C overnight.

**SAX-HPLC separation of disaccharides**

Enzymatic digests of oligosaccharides were diluted in 1 ml water and applied to ProPac PA1 SAX-HPLC column (4.6 mm × 250 mm; Dionex, Camberley, UK) running on an Agilent 1100 Series HPLC system. The column was pre-equilibrated in water. After sample loading the column was briefly washed with water and disaccharides were eluted using a 45 ml linear gradient of 0 – 1 M NaCl at a flow rate of 1 ml/min. Disaccharides were detected by in-line UV absorption at 232 nm. The elution time for each peak was compared to the elution times of different HS standards (Iduron).
