# Supplementary figures and images for: Synthetic Heparan Sulfate Oligosaccharides Inhibit Endothelial Cell Functions Essential for Angiogenesis
Source: PLoS One. 2010 Jul 21;5(7):e11644. doi: 10.1371/journal.pone.0011644 (PMC2908126; doi:10.1371/journal.pone.0011644)

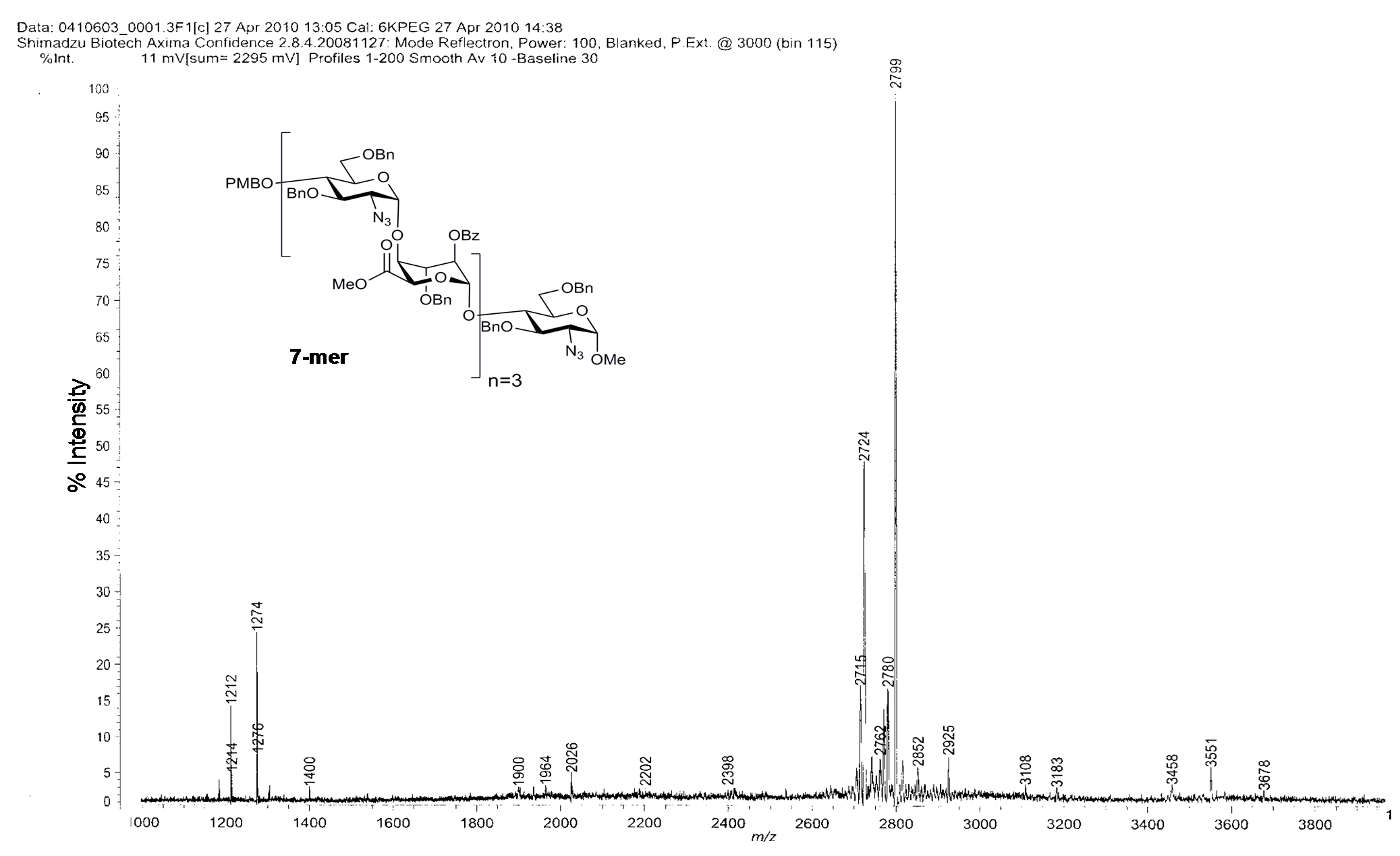

Supplement: Figure S1 — Full scan mass spectrum of protected intermediate 7-mer oligosaccharide. MALDI-TOF m/z calculated for [M+Na+] (C152H156N12O39Na+): 2797 (100.0%), 2798 (90.2%), 2796 (58.1%); found: 2799. (0.29 MB TIF) [file pone.0011644.s002.tif]

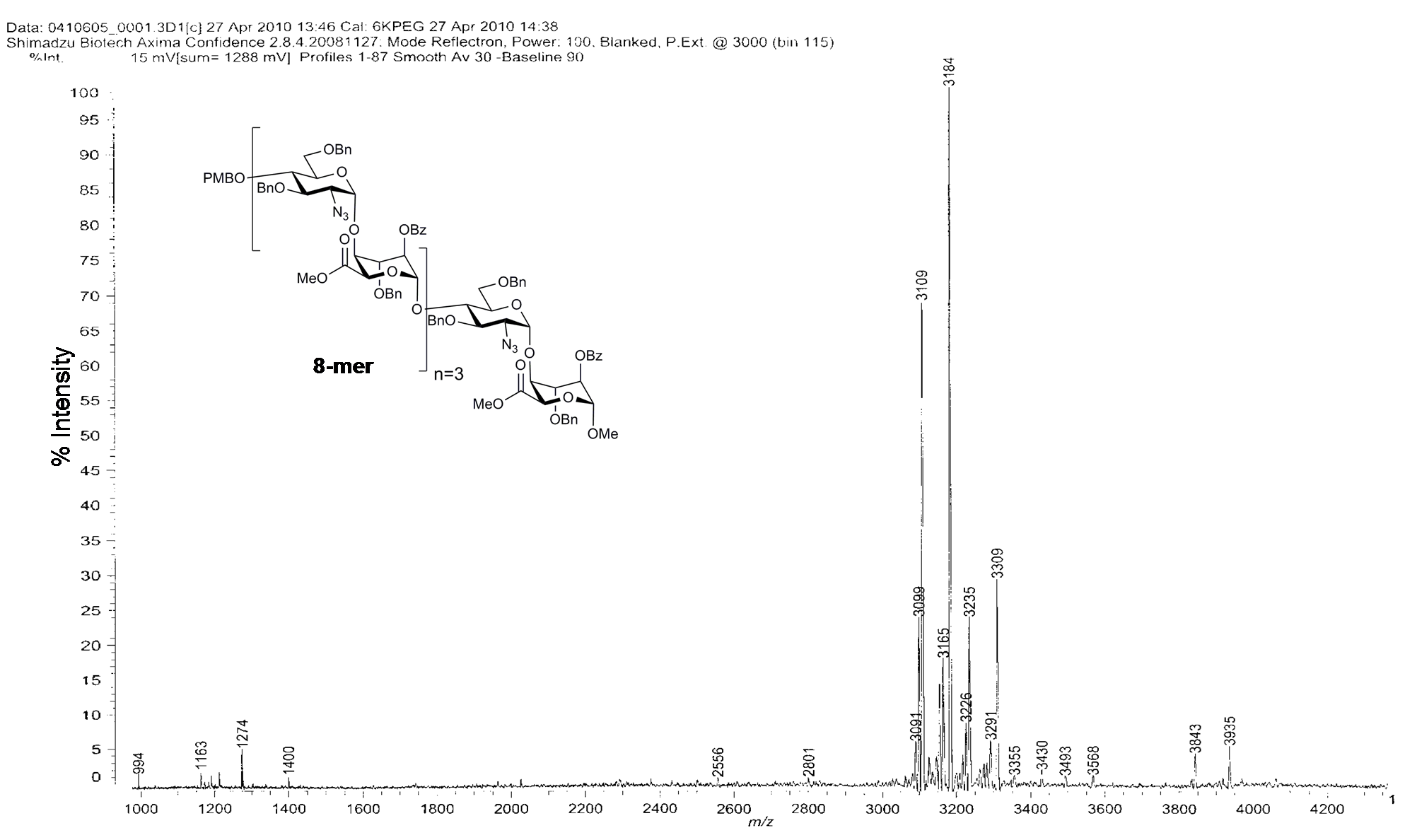

Supplement: Figure S2 — Full scan mass spectrum of protected intermediate 8-mer oligosaccharide. MALDI-TOF m/z calculated for [M+Na+] (C173H176N12O46Na+): 3182 (100.0%), 3181 (98.1%), 3183 (70.6%); found: 3184. (0.28 MB TIF) [file pone.0011644.s003.tif]

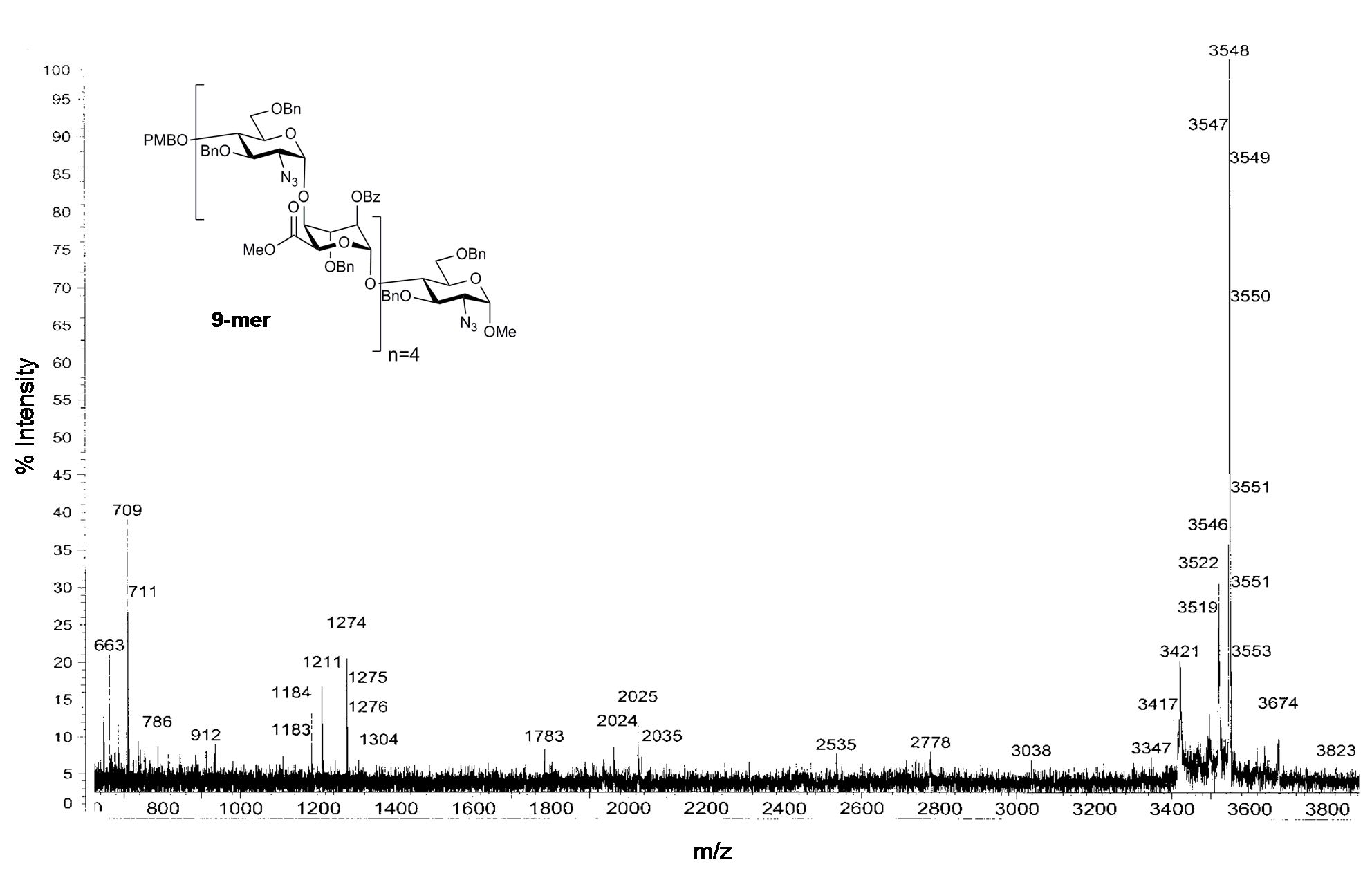

Supplement: Figure S3 — Full scan mass spectrum of protected intermediate 9-mer oligosaccharide. MALDI-TOF m/z calculated for [M+Na+] (C193H197N15O50Na+): 3549 (100.0%), 3548 (88.2%), 3550 (78.2%); found: 3548. (0.31 MB TIF) [file pone.0011644.s004.tif]

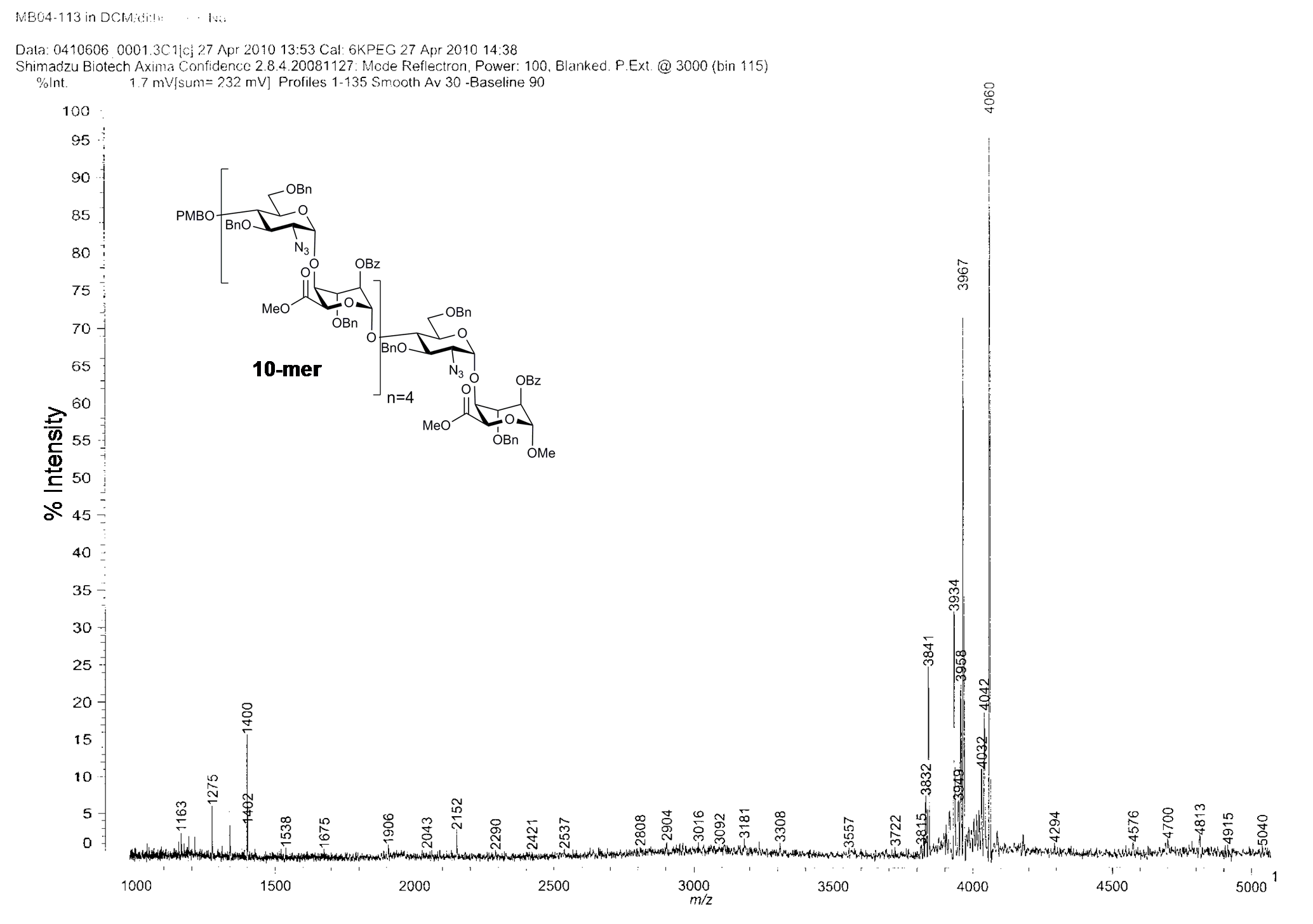

Supplement: Figure S4 — Full scan mass spectrum of protected intermediate 10-mer oligosaccharide. MALDI TOF m/z calculated for [M+Na+] (C214H217N15O57Na+): 3933 (100.0%), 3934 (84.3%), 3932 (81.4%); found: 3934. m/z calculated for [M-H+I+Na+] (C214H216IN15O57Na+): 4059 (100.0%), 4060 (84.3%), 4058 (81.4%); found: 4060. (0.35 MB TIF) [file pone.0011644.s005.tif]

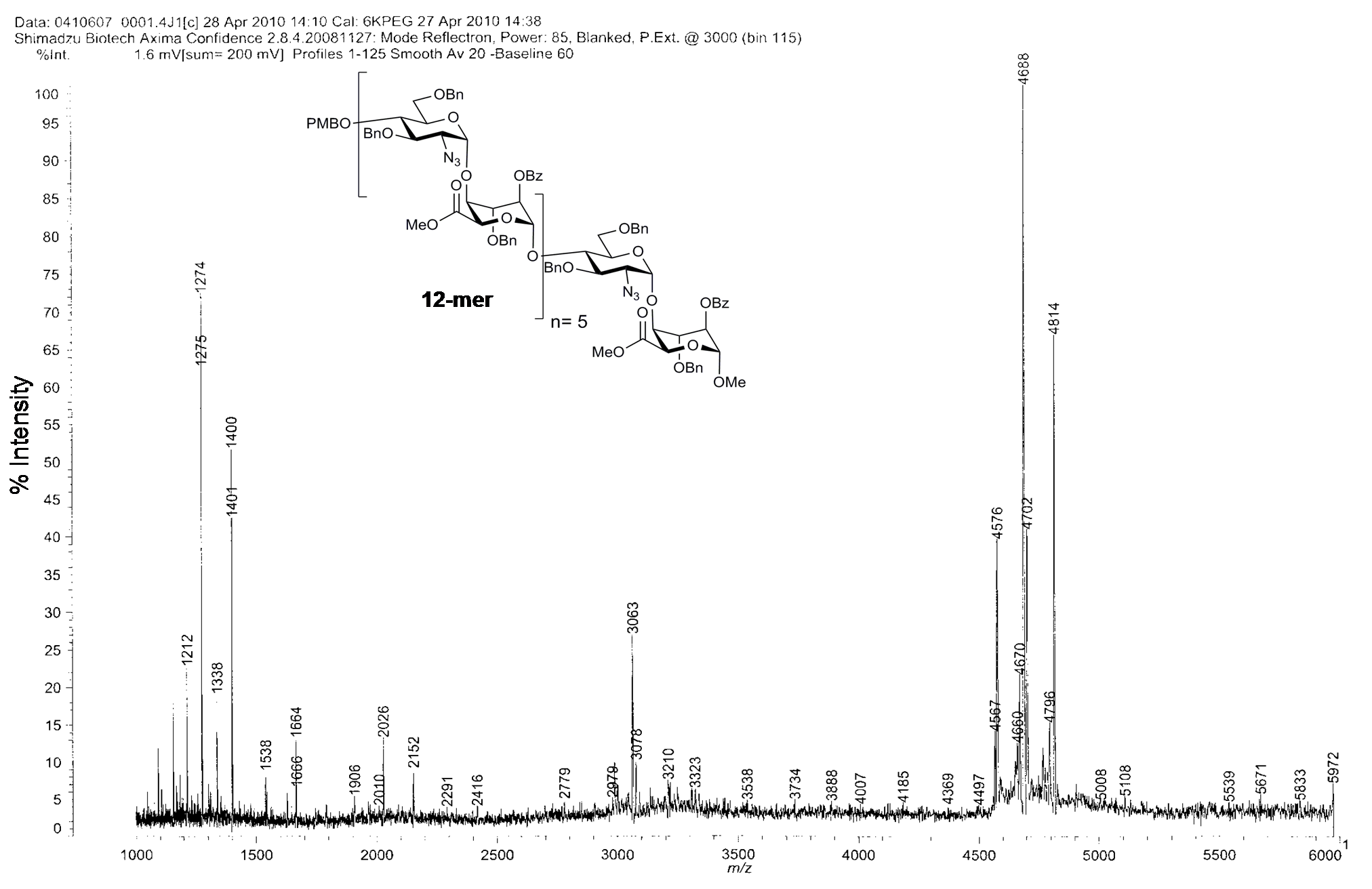

Supplement: Figure S5 — Full scan mass spectrum of protected intermediate 12-mer oligosaccharide. MALDI TOF: m/z calculated for [M+Na+] (C255H258N18O68Na+): 4685 (100.0%), 4686 (99.5%), 4687 (75.4%); found: 4688. m/z calculated for [M-H+I+Na+] (C255H257IN18O68Na+): 4811 (100.0%), 4812 (99.5%), 4813 (75.4%); found: 4814. (0.38 MB TIF) [file pone.0011644.s006.tif]

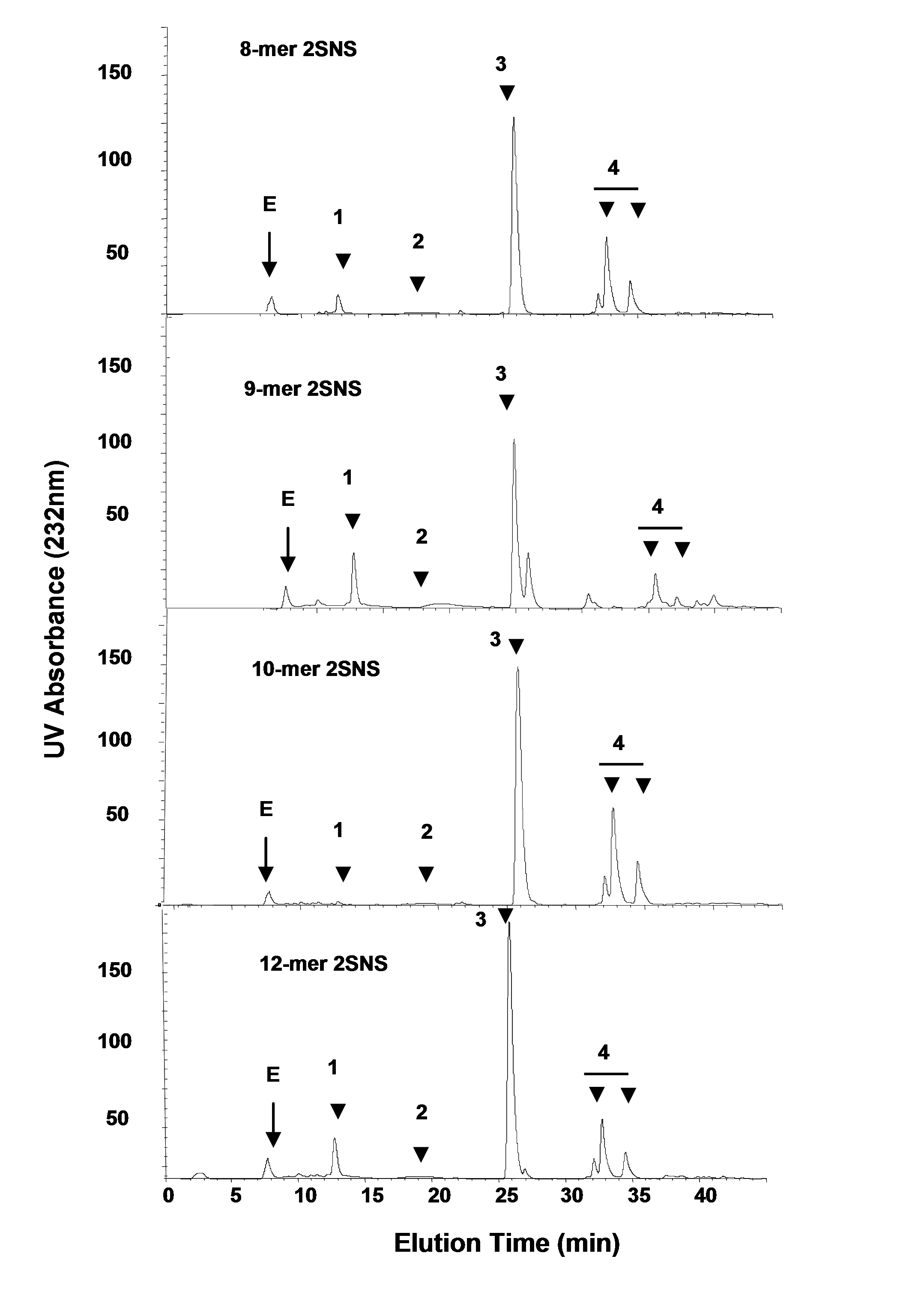

Supplement: Figure S6 — Disaccharide analysis of 8-mer 2SNS, 9-mer 2SNS, 10-mer 2SNS and 12-mer 2SNS oligosaccharides. Separation of disaccharides by SAX-HPLC is shown. Arrowheads show elution positions for UA-GlcNS (1), UA(2S)-GlcNAc (2), UA(2S)-GlcNS (3) and tetrasaccharides (4) as determined by comparison with elution times of HS standards. UA - uronic acid; GlcNAc - N-acetylated glucosamine; GlcNS - N-sulfated glucosamine; 2S - 2-O sulfate; E - enzymes. (0.25 MB TIF) [file pone.0011644.s007.tif]

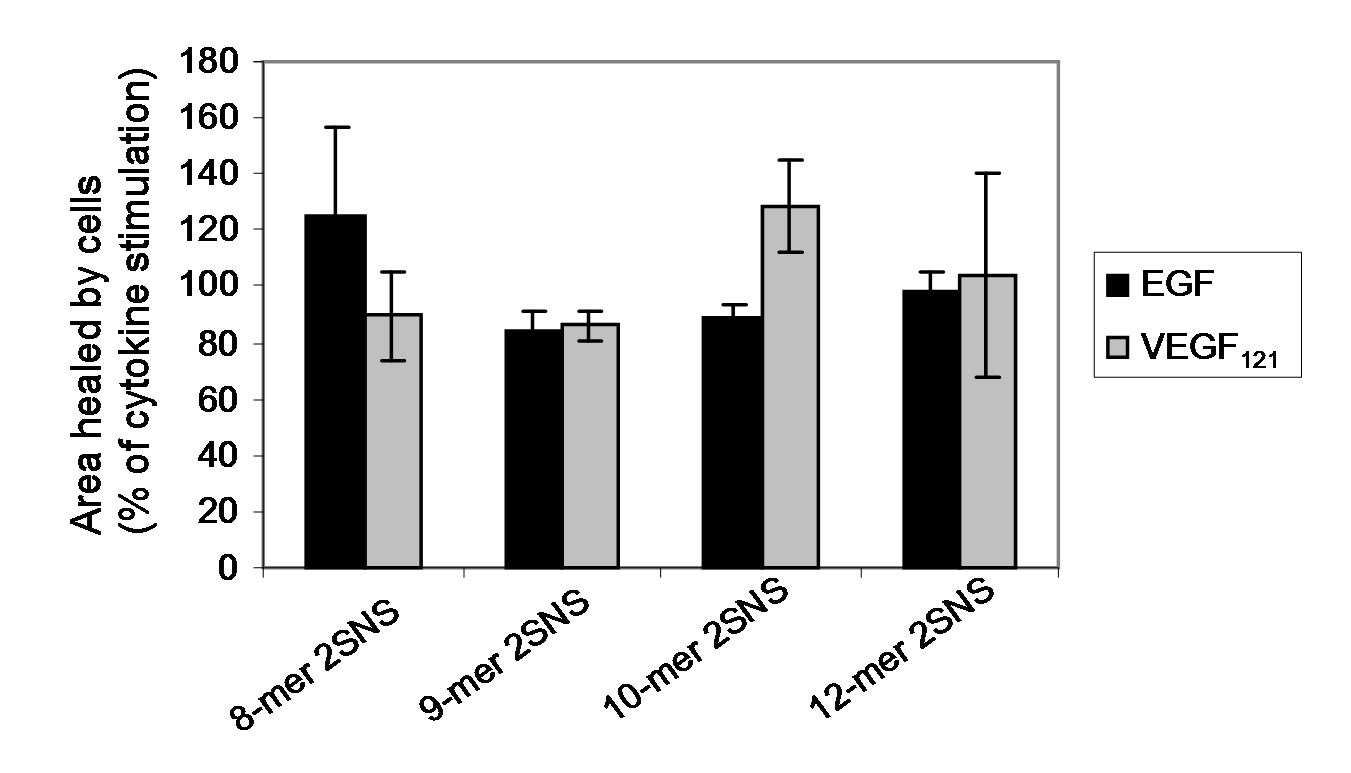

Supplement: Figure S7 — Biologically active 2SNS oligosaccharides that inhibit FGF2-induced cell migration have no effect on EGF- and VEGF121-stimulated cell advancement. Confluent layers of serum-starved immortalized HUVECs were wounded, EGF (20 ng/ml) or VEGF121 (20 ng/ml) were added to stimulate cell migration into the wound in the absence or presence of 8-mer 2SNS, 9-mer 2SNS, 10-mer 2SNS and 12-mer 2SNS oligosaccharides dosed at 50 mg/ml concentration. The wound area at baseline and after 24 hours was measured. The area that healed in the presence of cytokines alone when compared to serum-starved cells is expressed as 100%. The effect of oligosaccharides is expressed as percentage of repopulated area by cells stimulated with the cytokine alone. Data is presented as the mean ± SD (n = 3). (0.09 MB TIF) [file pone.0011644.s008.tif]
