# Supplementary material for: Synthetic Heparan Sulfate Oligosaccharides Inhibit Endothelial Cell Functions Essential for Angiogenesis
Source: PLoS One. 2010 Jul 21;5(7):e11644. doi: 10.1371/journal.pone.0011644 (PMC2908126; doi:10.1371/journal.pone.0011644)
Supplement: Table S1 — Disaccharide composition of 2SNS oligosaccharides. (0.03 MB DOC) [file pone.0011644.s009.doc]

| **Oligosaccharide** | **UA-GlcNS** | **UA(2S)-GlcNS** | **Tetrasaccharides** |
| --- | --- | --- | --- |
| 8-mer 2SNS | 3.0% | 75.5% | 21.5% |
| 9-mer 2SNS | 11% | 79.6% | 9.6% |
| 10-mer 2SNS | 0% | 79.4% | 20.6% |
| 12-mer 2SNS | 6.7% | 80.5% | 12.7% |

**Table S1.** Disaccharide composition of 2SNS oligosaccharides.

UA – uronic acid; GlcNS - *N*-sulfated glucosamine; 2S - 2-*O*-sulfate.
